# Characterising and Predicting Haploinsufficiency in the Human Genome

**DOI:** 10.1371/journal.pgen.1001154

**Published:** 2010-10-14

**Authors:** Ni Huang, Insuk Lee, Edward M. Marcotte, Matthew E. Hurles

**Affiliations:** 1 Wellcome Trust Sanger Institute, Wellcome Trust Genome Campus, Cambridge, United Kingdom; 2 Center for Systems and Synthetic Biology, Department of Chemistry and Biochemistry, Institute for Cellular and Molecular Biology, University of Texas, Austin, Texas, United States of America; 3 Department of Biotechnology, College of Life Science and Biotechnology, Yonsei University, Seoul, South Korea; University of Aarhus, Denmark

## Abstract

Haploinsufficiency, wherein a single functional copy of a gene is insufficient to maintain normal function, is a major cause of dominant disease. Human disease studies have identified several hundred haploinsufficient (HI) genes. We have compiled a map of 1,079 haplosufficient (HS) genes by systematic identification of genes unambiguously and repeatedly compromised by copy number variation among 8,458 apparently healthy individuals and contrasted the genomic, evolutionary, functional, and network properties between these HS genes and known HI genes. We found that HI genes are typically longer and have more conserved coding sequences and promoters than HS genes. HI genes exhibit higher levels of expression during early development and greater tissue specificity. Moreover, within a probabilistic human functional interaction network HI genes have more interaction partners and greater network proximity to other known HI genes. We built a predictive model on the basis of these differences and annotated 12,443 genes with their predicted probability of being haploinsufficient. We validated these predictions of haploinsufficiency by demonstrating that genes with a high predicted probability of exhibiting haploinsufficiency are enriched among genes implicated in human dominant diseases and among genes causing abnormal phenotypes in heterozygous knockout mice. We have transformed these gene-based haploinsufficiency predictions into haploinsufficiency scores for genic deletions, which we demonstrate to better discriminate between pathogenic and benign deletions than consideration of the deletion size or numbers of genes deleted. These robust predictions of haploinsufficiency support clinical interpretation of novel loss-of-function variants and prioritization of variants and genes for follow-up studies.

## Author Summary

Humans, like most complex organisms, have two copies of most genes in their genome, one from the mother and one from the father. This redundancy provides a back-up copy for most genes, should one copy be lost through mutation. For a minority of genes, one functional copy is not enough to sustain normal human function, and mutations causing the loss of function of one of the copies of such genes are a major cause of childhood developmental diseases. Over the past 20 years medical geneticists have identified over 300 such genes, but it is not known how many of the 22,000 genes in our genome may also be sensitive to gene loss. By comparing these ∼300 genes known to be sensitive to gene loss with over 1,000 genes where loss of a single copy does not result in disease, we have identified some key evolutionary and functional similarities between genes sensitive to loss of a single copy. We have used these similarities to predict for most genes in the genome, whether loss of a single copy is likely to result in disease. These predictions will help in the interpretation of mutations seen in patients.

## Introduction

With array-based copy number detection and the current generation of sequencing technologies, our ability to discover genetic variation is running far ahead of our ability to interpret the functional impact of that variation. Several software tools exist for predicting the phenotypic impact of mutations that change the amino acid sequence of an encoded protein [1]. These tools are essentially proteomic and genomic, rather than genetic, in perspective; no distinction is made between mutations that are dominant or recessive in action. By contrast, there is a lack of tools that predict the phenotypic impact at the organismal level of unambiguous loss-of-function mutations of an encoded protein (e.g. truncating mutations and whole gene deletions). Not all loss-of-function mutations are deleterious, especially when heterozygous. It is generally considered that recessivity is the norm for diploid genomes [2]. Some loss-of-function mutations even confer selective advantages [3]. It is clear from resequenced exomes [4] and genomes [5] and CNV surveys [6] that every genome harbours tens of unambiguous loss-of-function mutations.

A pressing clinical need for interpreting genetic variation is in distinguishing between pathogenic and benign copy number variants (CNVs) revealed by array-based profiling of patients [7]. With the current resolution of microarrays in clinical practice, these variants are typically large, rare deletions, often encompassing multiple genes. The most obvious pathogenic mechanism for heterozygous loss-of-function mutations (such as large rare deletions) is haploinsufficiency (HI), wherein a single functional copy of a gene is insufficient to maintain normal function. Only a few hundred genes have been reported haploinsufficient so far [8], [9]. Previous studies have shown that gene sets related to haploinsufficiency, such as genes implicated in dominant diseases and genes overlapped by CNVs, have biased evolutionary and functional properties [10]–[12]. However, such investigations often compare those gene sets to the genome average and have been descriptive rather than predictive in scope.

We sought to explore further systematic biases in the properties of known HI genes, and to develop a predictive model to assess for each gene in the genome the probability that it exhibits haploinsufficiency with respect to the severe developmental disorders that are the mainstay of clinical genetic practice. As it is not known for most genes in the genome whether or not they exhibit haploinsufficiency, we maximized the power of this predictive approach by assembling a large training set of ‘haplosufficient’ (HS) genes that do not exhibit haploinsufficiency resulting in severe developmental anomalies. We reasoned that currently the most effective way of screening for HS genes is use robust CNV discovery to identify genes that are wholly or partially deleted among thousands of adults recruited as controls for genome-wide association studies. We take advantage of the fact that the impact of large deletions on coding sequence is more unambiguously interpretable than other types of genetic variation, such as point mutations or small insertion/deletions.

## Results

**Figure 1:**
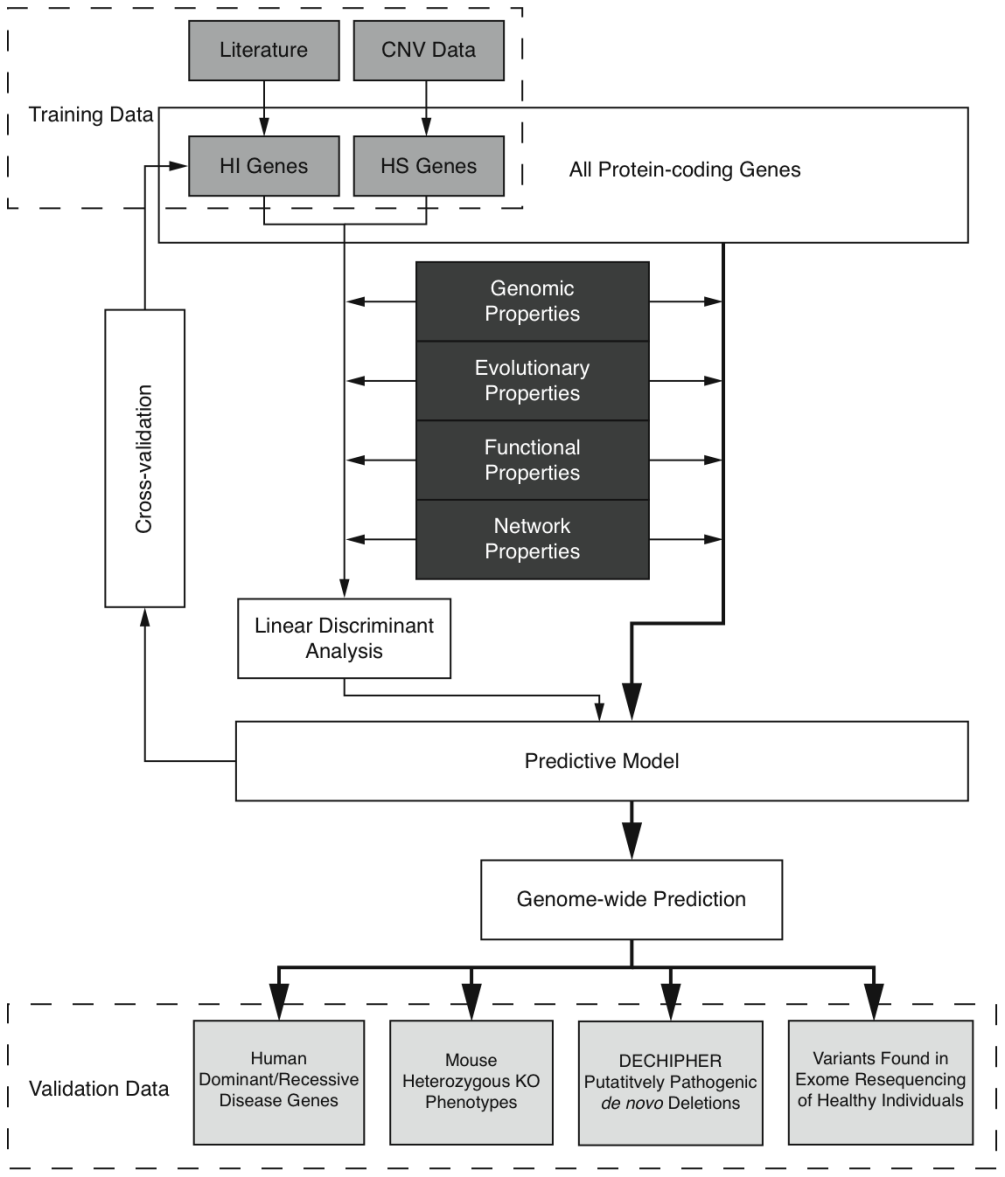
Outline of the prediction framework.

Here we predict human haploinsufficient genes by integrating diverse genomic, evolutionary and function properties that we show are characteristic of haploinsufficiency. We further validate those predictions with independent experimental and clinical data. The framework of this study is outlined in Figure 1. While various, but not necessarily mutually exclusive, physiological mechanisms have been proposed to underlie haploinsufficiency, including dosage threshold effects and altered stoichiometry of a macromolecular complex [13], [14], our approach does not assume that one or other of these mechanisms predominates.

We first compiled a list of known human HI genes and a catalog of HS genes. Known HI genes were collated from literature [8], [9]. The catalog of HS genes was generated from genes disrupted in a loss-of-function manner in control individuals used in genome-wide association studies by CNVs detected in data from the Affymetrix 6.0 chip (see Methods and Figure S1). We identified 2,676 putative HS genes seen in any control individuals and 1,079 seen in two or more controls (Table S1 and Figure S2), and used the latter set in most downstream analyses Thus the final list of HI and HS genes contains 301 and 1,079 genes respectively.

**Figure 2:**
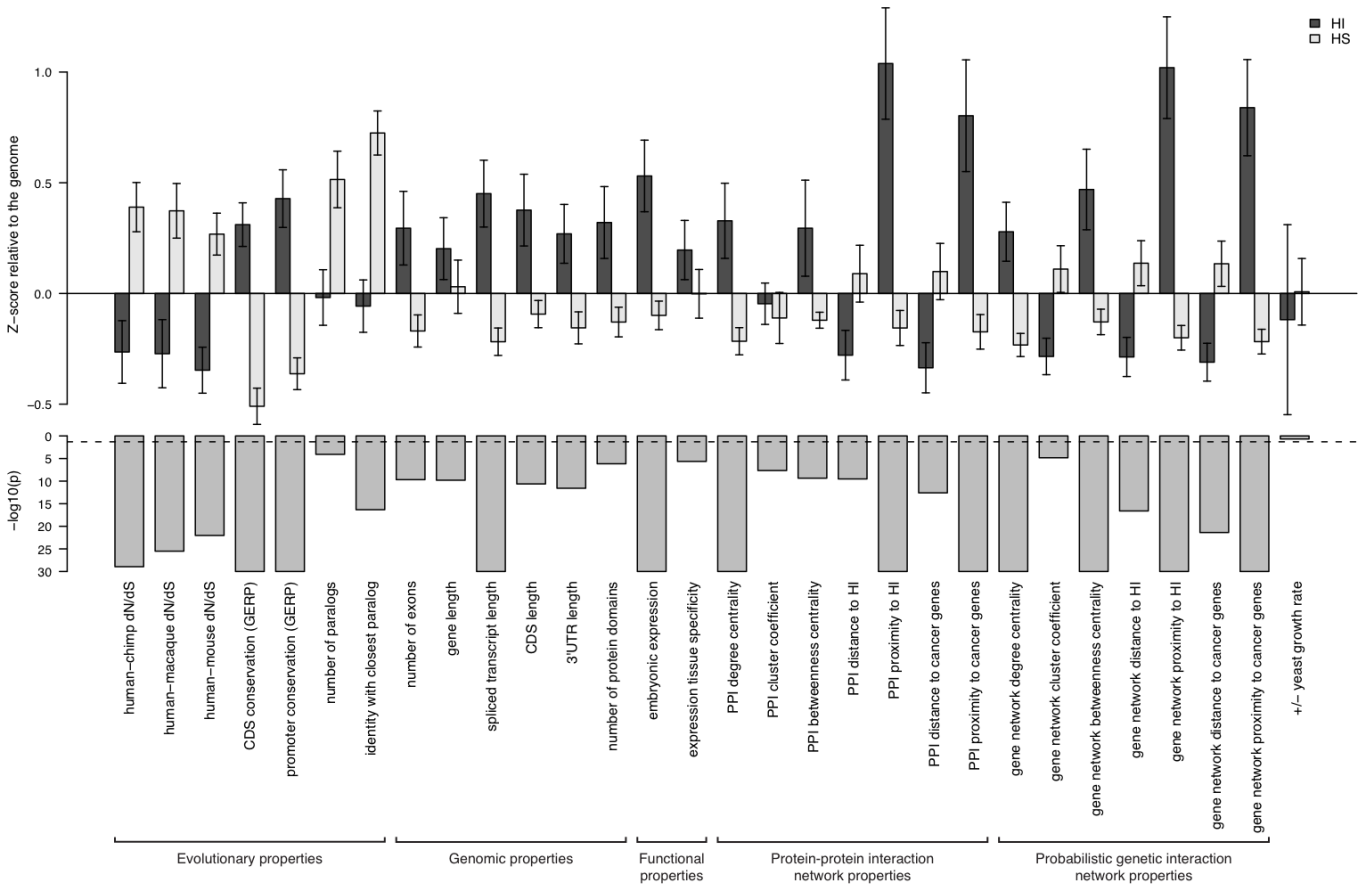
Properties that distinguish HI genes from HS genes. The upper part of the figure shows the comparison of the mean of each individual property between HI genes and HS genes. The values are transformed to z-scores relative to the genome average. The error bars represent two times the standard error of the mean. The bars in the middle part shows the transformed p value (−log_10_(p)) of the Mann-Whitney U test on each property. The dashed line marks a p value of 0.05.

To systematically assess the difference in properties between HI and HS genes, we gathered a large number of annotations describing the evolutionary, functionary and interaction properties of genes (see Methods) and examined the distribution of each individual property in HI and HS genes. We found that HI genes have consistently more conserved coding sequence (human-macaque dN/dS, p = 3.12e-26), less mutable promoter (p<1e-30), paralogs with lower sequence similarity (p = 1.84e-9), longer spliced transcript (p<1e-30), longer 3′UTR (p = 2.63e-12), higher expression during early development (p = 1.10e-15), higher tissue specificity in expression (p = 2.29e-6), more interaction partners in both a protein-protein interaction network (p<1e-30) and a gene interaction network (p<1e-30) and higher chances of interacting with other known HI genes (p<1e-30) and cancer genes (p<1e-30) (Figure 2). Some biological insights can be gained from these comparisons. For instance, the average sequence identity to the closest paralog of HS genes is significantly higher than that of HI genes, suggesting functional compensation between recently duplicated genes that shield each other from reduction in dosage. Interestingly, growth rate of yeast heterozygous deletion strains does not seem to differ between their HI human homologs and HS human homologs, probably reflecting the vast functional differences between the majority of yeast and human genes, except those involved in highly conserved cellular processes.

The highly significant differences in genomic, evolutionary, functional and network properties between HI and HS genes suggest they may be predictive of haploinsufficiency. However, since each annotation is only available for a fraction of genes in the genome (Table S2), there is a trade-off between the increase in prediction performance by considering more properties and the decrease in the genomic coverage of genes we could predict. Therefore, we aimed to select a small number of most predictive properties that are relatively ‘orthogonal’ in the kind of information they provide (Methods and Table S3). After evaluating many different possible combinations of predictor variables (Figure S3) we selected a model comprising of dN/dS between human and macaque, promoter conservation, embryonic expression and network proximity to known HI genes. The training data with all these information available consisted of 234 HI genes and 326 HS genes.

**Figure 3:**
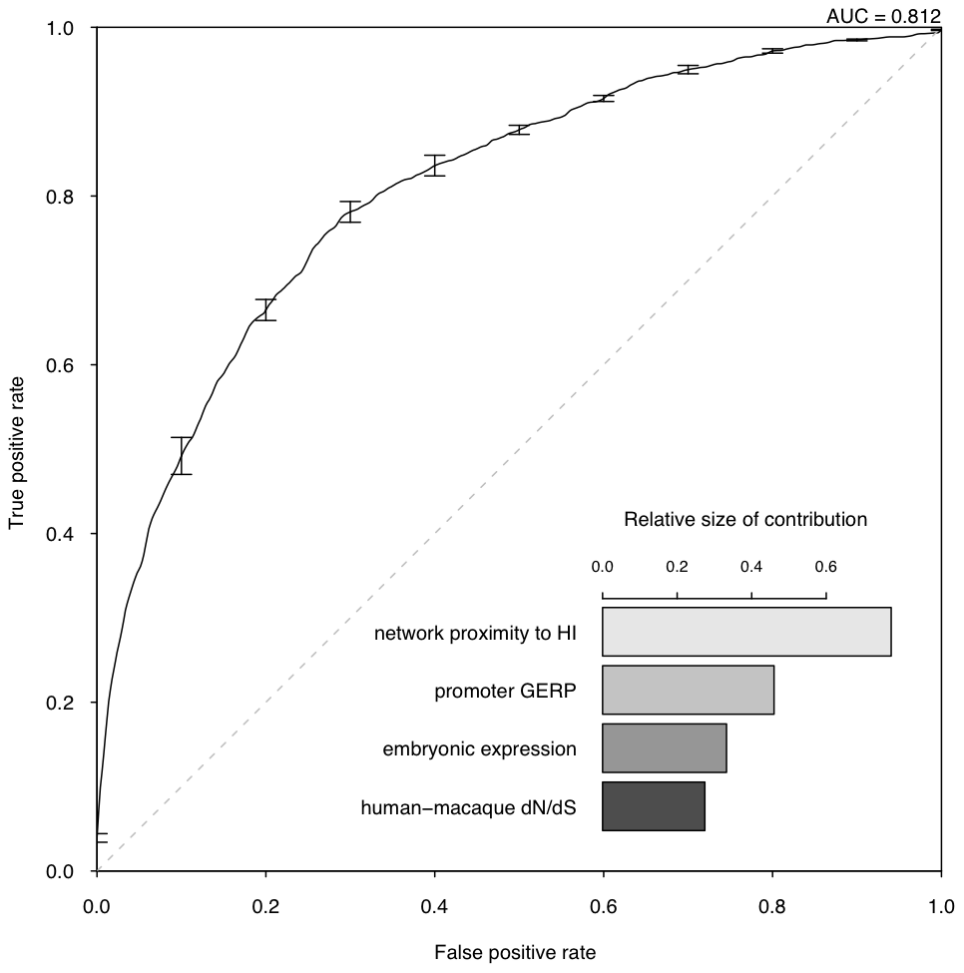
Assessment of model performance. The ROC curve demonstrates the performance of the model evaluated by 10-fold cross-validation. The lower right part shows the relative contribution of each predictor variable to the prediction model measured by the absolute value of the scaling factor of each predictor variable constituting the linear discriminant.

We are interested in the relative contributions of each selected property in the model. We used linear discriminant analysis (LDA) as the supervised classifier, which, given multi-dimensional data and class labels, finds the linear combination of the given dimensions (linear discriminant) that maximizes the inter-class variance. We scaled each property to the same variance before entering the model so that their contribution can be measured by the coefficients of the resulting linear discriminant (Figure 3). We found that proximity to known HI genes in the probabilistic gene network is the single most heavily weighted predictor of haploinsufficiency.

To evaluate the performance of the model, we adopted a 10 fold cross-validation strategy and calculated the area under the ROC curve (AUC) (Figure 3) and Matthew correlation coefficient (MCC) (Methods) as measurements of prediction performance. The model achieved an AUC of 0.81 and a MCC of 0.50. We showed that this prediction accuracy is not appreciably affected by the calling threshold used to define the CNVs that underpin the HS gene catalog, nor by the frequency threshold in controls used to define HS genes (Figure S6). We also showed that prediction is not appreciably improved by using a Support Vector Machine classifier, which is more computationally intensive (Figure S7). We demonstrated that combining the predictor variables together generated a more predictive model than considering any of the individual predictor variables in isolation (max AUC = 0.78 for network proximity to known HI genes, Figure S4). We also confirmed our hypothesis that contrasting known HI and inferred HS genes should be more predictive of HI than simply contrasting known HI genes to the rest of the genome (max AUC = 0.75, Figure S5).

**Figure 4:**
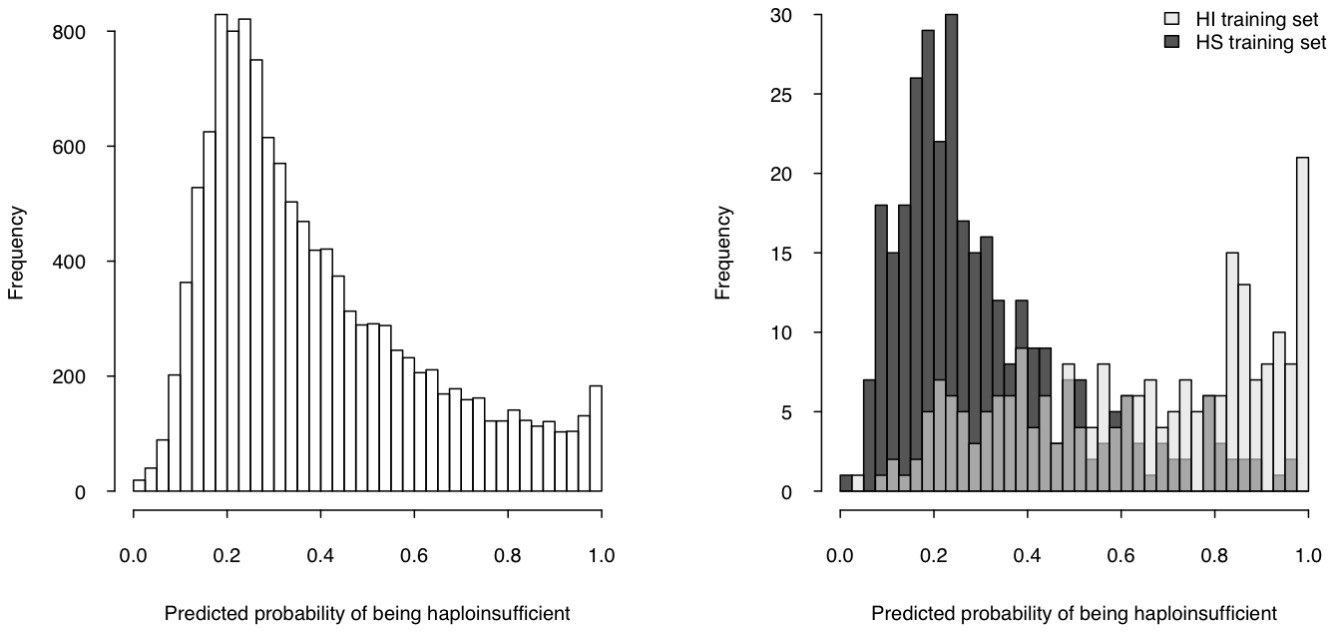
Predicted probability of being haploinsufficient across the genome. The histogram on the left shows the distribution of the predicted probability of being haploinsufficient ( p(HI) )of all 12,443 predictable genes. The histograms on the right shows the distribution of the predicted p(HI) of the HI training set (light grey) and the HS training set (dark grey).

We then used the model to estimate a probability of being HI ( p(HI) ) for all protein-coding genes in the genome for which all four selected properties were available (12,443 genes, over half of the total protein-coding genes) (Table S5, Dataset S1), the distribution of which is clearly bimodal, with a large peak near 0.2 and a much smaller peak at 1 (Figure 4, left). The distributions of p(HI) for the HI and HS training sets are clearly differentiated (Figure 4, right). It is not possible to assess how well-calibrated these probabilities are, as the fraction of human genes that exhibit HI is not known. We therefore sought to validate these predictions using indirect approaches that examined the distribution of p(HI) in independent gene sets enriched for HI. As there is no credible estimation of the number of human HI genes, in some of the following validation analyses we arbitrarily labeled the genes in the top 10% of p(HI) as being predicted HI genes. However, the results were robust against this threshold being varied by at least a factor of at least 2 (data not shown).

**Figure 5:**
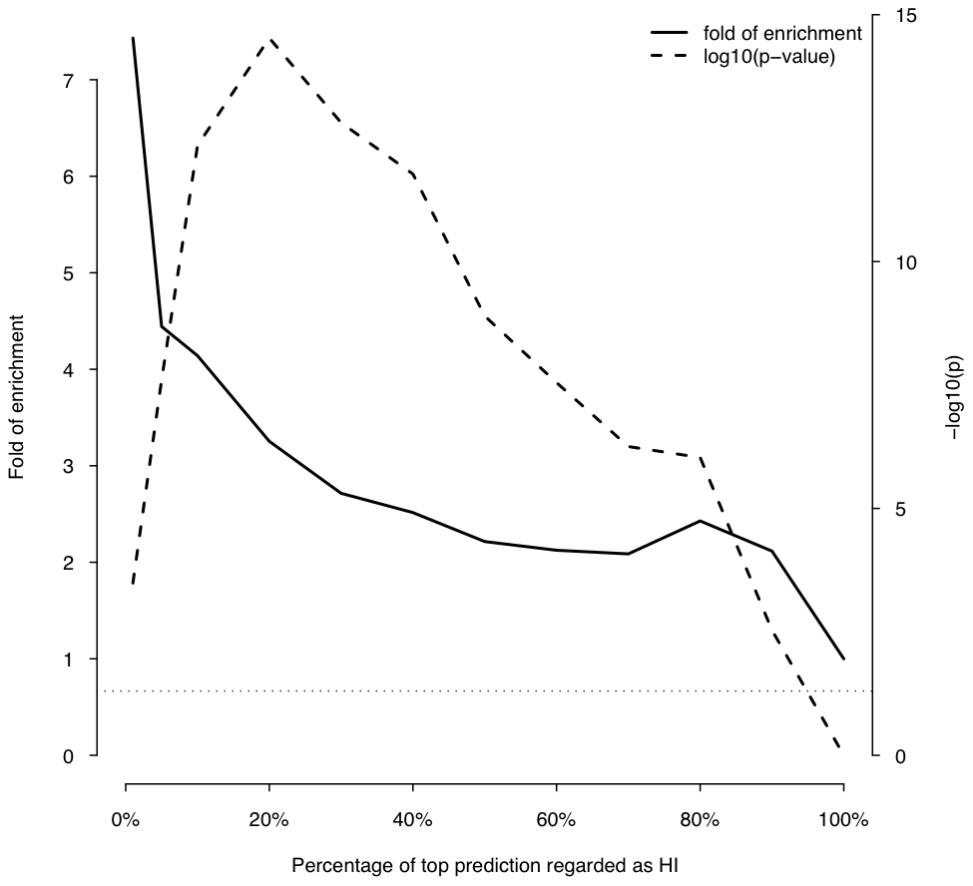
Enrichment of predicted HI genes in dominant genes relative to recessive genes. This plot shows the fold of enrichment of predicted HI genes in dominant genes relative to recessive genes (thick solid line) as a function of the proportion of top predictions labeled as being haploinsufficient. Also plotted is the transformed p value (−log_10_(p)) of the corresponding Fisher's exact test (thick dashed line). The horizontal dashed line marks the p value of 0.05.

**Figure 6:**
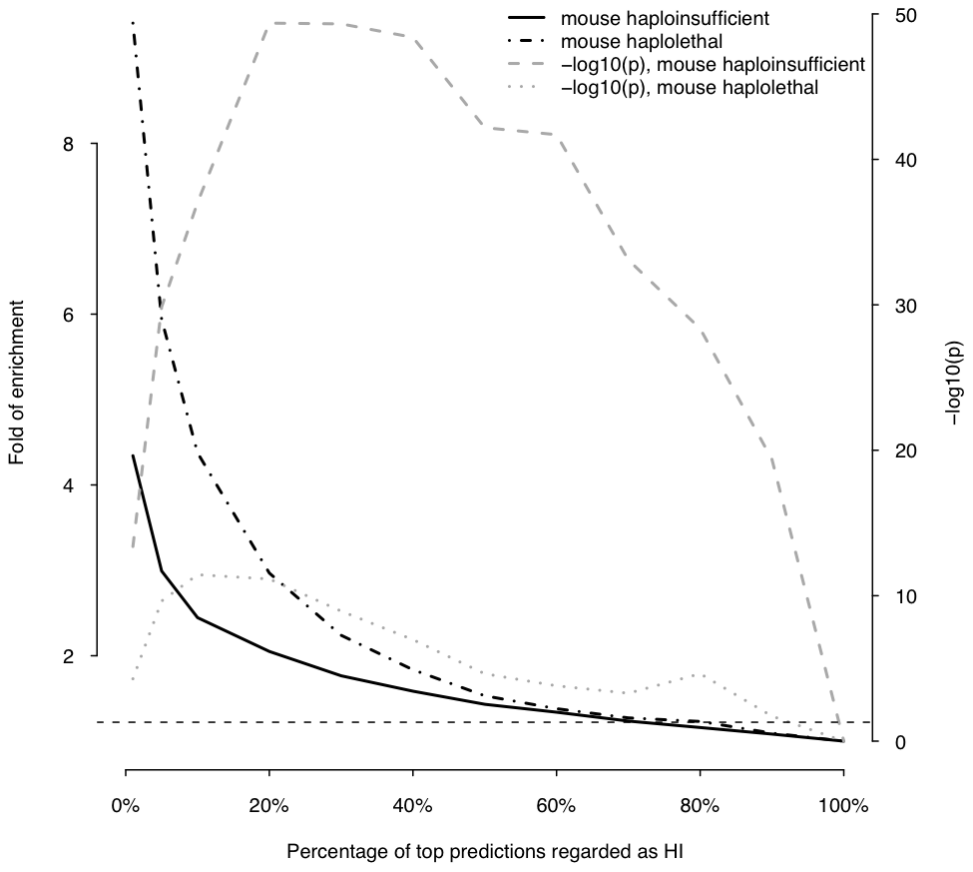
Enrichment of predicted HI genes in orthologs of mouse haploinsufficient genes and mouse haplolethal genes. This plot shows the fold of enrichment of predicted HI genes in human orthologs of mouse haploinsufficient genes (black solid line) and mouse haplolethal genes (black dashed line) relative to the genome average as a function of the proportion of top predictions labeled as being haploinsufficient. The two lines in grey show the transformed p values of the corresponding Fishers' exact test. The horizontal dashed line marks the p value of 0.05.

First, we asked if genes implicated in human dominant diseases were enriched in our predicted HI genes relative to recessive-disease-causing genes. We retrieved 571 and 772 genes implicated in dominant and recessive disease from the OMIM and hOMIM database [10], [15], respectively, with no information regarding haploinsufficiency (and thus not included in our training data), and compared the distribution of predicted p(HI) against each other. The HI status could be predicted for 392 dominant genes and 606 recessive genes, of which 87 and 39 were predicted as being HI, respectively. This 4.14 fold enrichment is highly significant (p = 4.46e-13, Fisher's exact test). Simply comparing the distribution of p(HI) values for these dominant and recessive genes also shows a highly significant shift towards high p(HI) values in dominant relative to recessive genes (p = 4.44e-16, Mann-Whitney U test; Figure 5). Second, we asked if heterozygous knockout of the orthologs of predicted human HI genes are more likely to cause severe phenotypic abnormalities in mice. For this purpose, we extracted a list of 1,523 mouse genes whose heterozygous knockout cause various abnormal phenotypes from MGI database [16], mapped them onto orthologous genes in humans, removed orthologs to genes in our training genesets and extracted the predicted p(HI) for the remainder. HI status could be predicted for the orthologs of 1,063 of these genes and 260 (24.5%) of them were predicted HI, indicating a 2.45 fold enrichment (p<1e-30, Fisher's exact test ) (Figure 6). If focusing on those genes of which the heterozygous LOF phenotypes involve prenatal lethality (MP:0002080), the fold of enrichment increased to 4.38 (p = 3.60e-12, Fisher's exact test) (28 predicted as HI out of 64 that could be predicted).

### Using haploinsufficient gene predictions to assess pathogenicity of deletions

**Figure 7:**
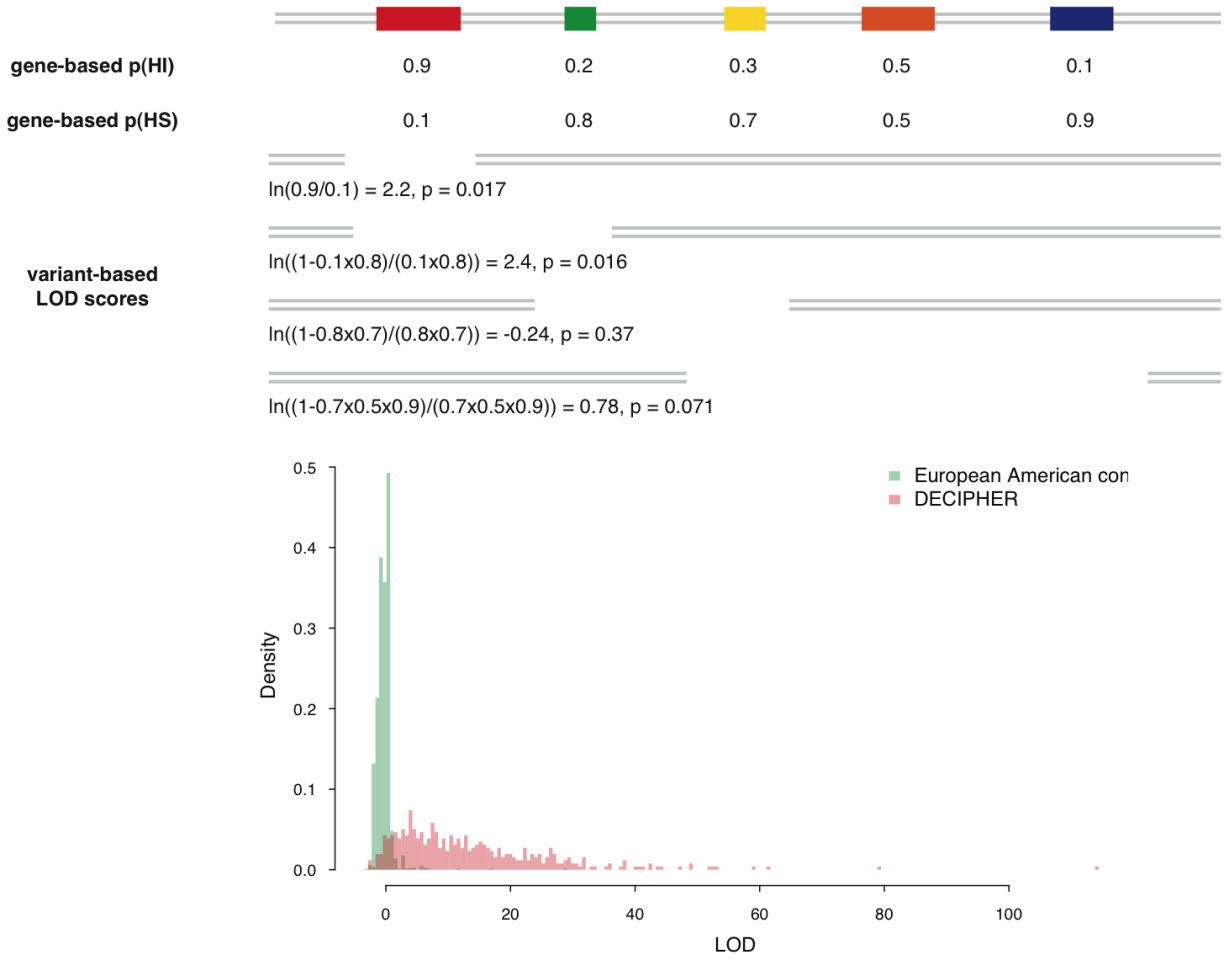
Calculation of deletion-based LOD scores and the distribution of LOD score of control individuals and pathogenic *de novo* deletions. The upper portion of the figure is a schematic demonstration of the calculation of the deletion-based LOD score. The contribution of genes with high p(HI) is accordingly weighted in a probabilistic way. The deletion with the largest LOD score in each individual is recorded and their distribution is shown in the lower portion of the figure. The distribution of maximal LOD scores of 2,322 control individuals are shown in green and the distribution of LOD scores of 487 pathogenic *de novo* deletions from DECIPHER are in red. Using the control distribution as the null, the probability a deletion is pathogenic can be assessed.

We investigated how our gene-based predictions of haploinsufficiency might be used to discriminate between benign and pathogenic genic deletions. We considered that a natural way to score the probability of a deletion causing a haploinsufficiency phenotype is to generate a LOD (log-odds) score comparing the probability that none of the genes covered by the deletion will cause haploinsufficiency with the probability that at least one of the genes will cause haploinsufficiency, as shown schematically in Figure 7. This LOD score is calculated using the formula below:
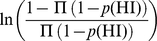
Higher LOD scores indicate deletions that are more likely to be pathogenic as a result of haploinsufficiency. Note that this score assumes that there is no statistical interaction between the genes.

We then considered how these deletion-based haploinsufficiency scores might be used to assess whether a genic deletion observed in a patient might cause their disease. One way of framing probabilistically this intuitively simple question is to estimate the opposing probability, that the deletion is unrelated to the patient's disease status. We can relate this to the probability of drawing an individual at random from a healthy control population with a deletion at least as pathogenic as the deletion in the patient. We can estimate this probability empirically as the proportion of healthy controls with a genic deletion having the same or greater haploinsufficiency score.

To test this approach, and to avoid circular reasoning, we generated a set of gene haploinsufficiency predictions using a slightly smaller set of HS genes identified in a large subset of the GWAS controls. The performance of the HI predictions using the slightly smaller HS gene training data was very similar to that of the full predictive model described in the previous section, as assessed by ten-fold cross validation (Text S1). We then identified LOF deletions in the remainder (N = 2,322) of the GWAS controls [17], which had not been used to train the predictive model, and determined the distribution of the maximal deletion haploinsufficiency score (based on the new gene haploinsufficiency predictions) observed in each control individual. We investigated whether the distribution of maximal LOD scores is significantly different between European-American (E-A) and African-American (A-A) GWAS controls. We observed that there was not a significant difference in median haploinsufficiency scores in E-A and A-A populations (p = 0.71, Mann-Whitney U test), although the E-A controls have a slightly longer tail of more pathogenic deletions (e.g. a higher proportion of E-A controls have deletions with LOD scores greater than 3, Table S4). The 50%, 90%, 95% and 99% percentiles of the distribution for maximal LOD score for E-A and A-A controls cohorts are listed in Table 1.

**Table 1:** Percentiles of the distribution of maximal LOD scores seen in controls.

Test cohort name	Sample size	HS gene source	50%	90%	95%	99%
African-American controls (GAIN)	889	WTCCC2+HapMap1	−0.39	0.67	0.82	2.32
European-American controls (GAIN)	1433	WTCCC2+HapMap1	−0.39	0.24	0.82	3.24

This table reports the LOD score at different percentiles of the distribution of maximal LOD scores seen in healthy controls from two populations: European-Americans and African-Americans. “HS gene source” reports the control data used to assemble the HS gene list used in training the predictive model. Note that the training data is distinct from the controls used to generate the distribution of maximal LOD scores to prevent any bias towards underestimating the LOD score percentiles.

We calculated a LOD score for each of 487 pathogenic *de novo* deletions submitted by clinical geneticists to the DECIPHER database [18]. We focused exclusively on deletions known to be *de novo* variants, as we infer that their pathogenicity has been ascribed primarily on the basis of their inheritance status. The distributions of maximal LOD scores in GWAS controls and LOD scores of pathogenic DECIPHER deletions are shown in Figure 7. The pathogenic deletions have strikingly significantly higher LOD scores than deletions observed in GWAS controls (p<1e-30, Mann-Whitney U test). We observed that for 92% of the pathogenic deletions there was a probability of less than 5% of drawing an individual at random from our control population with a genic deletion of equal or greater LOD score, and for 83% of pathogenic deletions there was a less than 1% probability.

**Figure 8:**
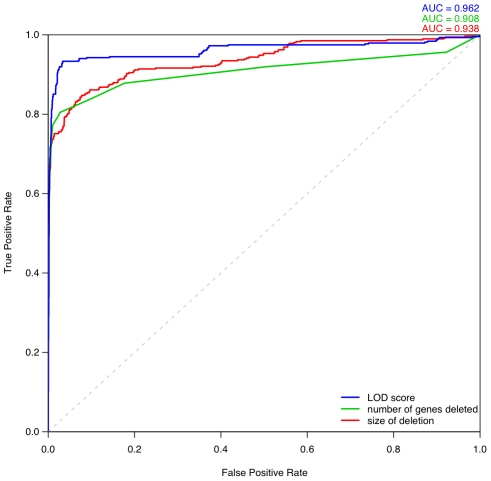
Comparison of different metrics for assessing deletion pathogenicity. Three ROC curves repesent the performance of three different methods for distinguishing between pathogenic deletions from DECIPHER and the most pathogenic deletions observed in control individuals. The blue curve denotes using LOD score calculated from predicted probability of exhibiting haploinsufficiency as the metric of pathogenicity. The green curve denotes using the number of genes deleted as the metric, in which case the most pathogenic deletion per individual is the one containing greatest number of genes in that individual. The red curve denotes using the size of deletion as the discriminating metric.

We computed ROC curves to compare three different approaches for discriminating between pathogenic deletions and deletions seen in controls: (i) our LOD scores, (ii) the length of the deletion, and (iii) the number of genes in the deletion (Figure 8). These ROC curves clearly show that the haploinsufficiency LOD score is the best metric for discriminating between pathogenic deletions in patients and deletions seen in controls. We provide a script and input files to calculate LOD scores and make comparisons with control data (Protocol S1).

### Haploinsufficient gene predictions and loss-of-function sequence variation

We investigated whether the gene-based probabilities of haploinsufficiency that we have generated are of general utility across different forms of genetic variation. If this is indeed the case then we should expect that genes harbouring loss-of-function substitutions or small indels in apparently healthy individuals should not have a high p(HI). We identified 349 genes as having LOF substitutions and indels in 12 recently sequenced exomes [4], of these, we could estimate p(HI) for 176 that were not also in the HS training set (and thus represent a fair set for independent comparisons). These genes are highly significantly enriched among genes with low probabilities of exhibiting haploinsufficiency (p = 1.06e-20 when comparing to the genome, and p<1e-30 when comparing to known HI genes, Mann-Whitney U test). This result implies that there are not substantial differences between genes that tolerate whole gene deletions and those that tolerate smaller loss-of-function variants. Moreover, by studying the allele frequency spectrum apparent in a large gene-resequencing dataset that has been extensively studied for patterns of selective constraint [19]–[21] we observed that nonsynonymous variants in genes more likely to exhibit haploinsufficiency are highly significantly skewed towards rarer variants than nonsynonymous variants in genes less likely to exhibit haploinsufficiency, in both African Americans and European Americans (p = 2.9e-7 and 4.0e-3 respectively, one-sided Mann-Whitney U test, see also Figure S9) reflecting greater, on average, selective constraint on genes we predict to exhibit haploinsufficiency (Text S1).

## Discussion

We have undertaken a systematic characterization of human haploinsufficient genes by contrasting them with a set of haplosufficient genes derived from non-pathogenic CNVs, developed a prediction model on the basis of the most significant differences, and assigned predicted probability of being haploinsufficient to more than half of the protein-coding genome.

Our finding that functional interaction with known HI genes was the single most predictive property of HI genes probably reflects the modularity of the interaction network, suggesting certain pathways or biological processes, such as early development morphogenesis, being more sensitive to dosage changes than others. However, it is also possibly influenced by an ascertainment bias with which HI genes are discovered.

The accuracy of our haploinsufficiency probabilties is limited by a number of factors, such as imperfect training data, although we have taken considerable steps to limit false positives, and missing data among predictor variables in our model. As network proximity to known HI genes is the single most predictive variable we eagerly await the construction of networks with increasing coverage and completeness, in the expectation that it should improve our prediction power. The gene coverage of our method could potentially be increased by using multiple imputation approaches to impute missing data [22]. To trial this approach, we imputed missing predictor variables using the predictive mean matching method, which allowed us to increase considerably the number of genes for which we could predict haploinsufficiency from 12,443 to 17,456 (Text S1, Table S5, Dataset S2). The resultant increase in size of training data also led to a slight improvement in prediction accuracy (AUC increased from 0.81 to 0.83, Figure S10), and we observed similar levels of enrichment of known dominant genes and mouse haploinsufficient genes pre- and post-imputation (Figure S11 and Figure S12), suggesting that multiple imputation is a reliable method to increase genome coverage. Another limitation of our method is the broad phenotypic outcome being predicted. Essentially, we are contrasting HI genes that cause a broad range of developmental disorders, with HS genes for which haploidy does not majorly impair an individual's ability to participate as a control in a genome-wide association study. We note that this broad phenotype is nevertheless considerably more constrained than that being considered by prediction algorithms based on evolutionary conservation, which are essentially integrating any deleterious phenotype manifested among any of the environments encountered during millions of years of evolution, across all possible modes of inheritance and genetic backgrounds. Despite the breadth of the phenotype implicit within conservation-based predictions, this class of algorithms has been demonstrated many times to be of appreciable utility [19], [23].

To support clinical interpretation of deletions seen in patients, we have transformed our gene-based predictions of haploinsufficiency into haploinsufficiency scores for individual deletions. Currently, clinicians typically use the length of a deletion or the number of genes deleted to assess the pathogenicity of a deletion. We have shown that our pathogenicity scores represent a superior metric to these existing approaches for classifying pathogenic deletions. We believe that the most appropriate use of these deletion-based haploinsufficiency scores is to compare deletions seen in patients with those seen in controls, and that quantifying the fraction of control individuals with a deletion at least as pathogenic as that seen in a patient provides a rational basis to classify pathogenic deletions. This fraction represents the probability of observing such a deletion by chance and thus the probability that a deletion will have been misclassified as pathogenic. A clinician can therefore set a particular haploinsufficiency score threshold to define pathogenic deletions through considering the misclassification rate with which they are comfortable. We have provided the necessary software tools to allow these haploinsufficiency scores to be calculated for any genic deletion, and automated calculation of these LOD scores and comparison with control deletions will be integrated into the forthcoming release (v5) of the DECIPHER database (personal communication: Helen Firth, Nigel Carter, Manual Corpas), which is used by over 170 clinical centres worldwide to interpret chromosomal rearrangements seen in patients (the gene-based predictions ( p(HI) ) are already available in the current release of DECIPHER, Figure S8).

We only observed subtle differences in the distribution of haploinsufficiency scores seen in European-American and African-American populations (Table S4), which might reflect a higher fraction of deleterious alleles in populations with non-African ancestry. Further investigation of these differences is warranted to see whether the haploinsufficiency scores observed in a patient ought to be compared with controls from a matched population.

It has recently been suggested that some developmental disorders result from the presence of two independent deletions in the same genome, the ‘two-hit’ hypothesis [24]. This hypothesis suggests a subtly different assessment of a patient's CNV data is required, through considering the question: ‘is the SET of deletions observed in my patient causal of their disease’. Another way of viewing this important question is that it requires consideration of the genome-wide haploinsufficiency ‘burden’ rather than the haploinsufficiency scores of individual deletions. The probabilistic framework we have established for assessing pathogenicity of individual deletions naturally extends to this situation. Rather than combining the haploinsufficiency probabilities for individual genes within a deletion to calculate a haploinsufficiency score for that variant, we can combine the haploinsufficiency probabilities for all deleted genes in the genome to calculate a haploinsufficiency LOD score for that genome, and compare this genome-wide haploinsufficiency score with those observed in healthy controls to assess the probability of sampling a healthy individual with a genome with a haploinsufficiency burden at least as high as that in the patient. This approach also naturally extends to an assessment of the genome-wide haploinsufficiency burden from other classes of LOF mutation.

One requirement of the haploinsufficiency score approach for assessing pathogenicity of individual deletions is that data quality between patients and controls is similar. If there are systematic differences between the sensitivity and specificity of CNV ascertainment in patients and controls then this may lead to biased comparisons of haploinsufficiency scores. This potential limitation is largely mitigated by an inherent focus on the largest deletions in a genome, which are typically long enough for many different technology platforms to have essentially complete sensitivity at very high specificities.

In addition to the use of the deletion-based and genome-wide haploinsufficiency scores described above, we envisage that our gene-based predictions of haploinsufficiency have two additional applications: (i) prioritization of variants for follow-up studies and (ii) integration into association testing to increase power; and we consider each of these these in turn. First, our predictions of haploinsufficiency provide a rational basis for prioritizing heterozygous variants for follow-up genetic and/or functional studies. The burden of having to validate increasing numbers of benign variants is an appreciable barrier to the translation into clinical practice of genomic technologies of ever-increasing resolution. A method that accurately hones in on potential causal variants could alleviate this burden considerably. The prioritization of variants need not be restricted solely to unambiguous loss-of-function variants. Most rare functional variants in any given genome are heterozygous nonsynonymous substitutions, many of which result in a complete or partial loss-of-function of the encoded gene. We contend that the prediction power of popular methods for predicting the functional impact of nonsynonymous substitutions from structural information and evolutionary conservation, such as SIFT [25] and POLYPHEN [26], is limited by an inability to discern from cross-species alignments whether purifying selection at a given site is acting in a recessive, additive or dominant manner. Combining these genotype-oblivious predictions with our predictions of haploinsufficiency, should enable rational, genotype-aware prioritization of heterozygous nonsynonymous variants. The second application of these predictions is to integrate them directly into association testing. It has been suggested that weighting variants by their probability of having a functional impact should improve power in resequencing studies to detect functional units (e.g. genes, pathways) enriched for functional variants [27]. As noted above, most of the rare variants considered in these studies are only observed in the heterozygous state, thus, if functional, they have to be exerting a dominant or semidominant effect, and predictions of haploinsufficiency, because haploinsufficiency is a major mechanism underlying dominance, are a highly relevant weighting factor.

The framework that we have developed that integrates functional, evolutionary and genomic properties of genes, could, by judicious selection of different training datasets be easily and broadly extended to include other classes of variant (e.g. duplications, gain of function mutations), different genetic models (e.g. recessive effects) and different, and potentially more specific, phenotypic outcomes (e.g. disease-specific).

## Methods

### Identifying haplosufficient genes from CNV data

We compiled a set of CNVs from three genotyping datasets generated from Affymetrix 6.0 platform, 210 unrelated HapMap individuals [28], 2,421 control individuals used in GWAS studies of bipolar and schizophrenia [17] and 6,000 individuals participating WTCCC2 as common controls, using Birdsuite [29]. All these CNVs were annotated against EnsEBML protein-coding gene annotation build 50 [30]. Genes with all transcripts satisfying one of the following criteria: deletion of over half of the coding sequence, deletion of the start codon, deletion of the first exon, deletion of splice-signal and deletion that causes frame-shift, were considered loss-of-function (Figure S1) and for the gene to be included as haplosufficient, such events are required to occur in at least two apparently healthy individuals.

### Preparation of predictor variables

#### Genomic properties

The length of gene, spliced transcript, 3′UTR and coding sequence and the number of exons were calculated on the basis of gene annotation downloaded from EnsEMBL. The number of protein domains was retrieved from EnsEMBL.

#### Evolutionary properties

dN/dS data was downloaded from EnsEMBL. Genomic Evolutionary Rate Profiling (GERP) [31] score was downloaded from EBI. Two summed GERP values, one for coding sequence and the other for promoter region, defined as bases within [−100, 100) window centered at transcription start site, were then calculated for all human protein-coding transcripts according to EnsEMBL annotations and summarized by gene using the median values. The number and identity of paralogs were downloaded from EnsEMBL.

#### Functional properties

Gene expression profiles in human were obtained from the GNF Atlas [32]. Total expression levels were normalized across genes and the standard deviation of expression across normal tissue types of each gene was used to indicate its tissue specificity of expression. Genes over-expressed by at least 8 fold in human embryonic stem cells [33], fetal tissues [32] and mouse fetal tissues [34] were collectively treated as genes expressed at embryonic stage. A binary coding was used to represent this property in which genes expressed at embryonic stage were labeled 1 and the rest were labeled 0.

#### Network properties

Two interaction networks were used. One is a binary protein-protein interaction network integrated from a number of sources [35]–[39]. The other is a probabilistic gene interaction network (a network of 470,217 links among 16,375 human genes calculated using methods previously described for yeast [40] and worm [41] and derived from 22 publicly available genomics datasets including DNA microarray data, protein-protein interactions, genetic interactions, literature mining, comparative genomics, and orthologous transfer of gene-gene functional associations from fly, worm, and yeast; I.L., E.M.M., manuscript in preparation) where the weight of a link is the log likelihood score of the interaction [40]. Measures of centrality (degree, betweenness) and modularity (cluster coefficient) were calculated using MCL [42]. Shortest path distance and sum of weight of interactions [41] were calculated as measures of proximity to a group of ‘seed’ genes.

#### Other properties

A list of 300 genes implicated in cancer was downloaded from the COSMIC database [43]. Growth rate of yeast heterozygous deletion strains were from Deutschbauer *et al* [13].

### Assessment of correlation of individual properties with haploinsufficiency

For continuous variables, the two-tailed Mann-Whitney U test was performed to assess if positive (haploinsufficient) and negative (haplosufficient) training data have the same median value for potential predictor variables. For two-class categorical features, Fisher's exact tests were performed. Statistical tests were performed using R (http://www.r-project.org).

### Feature selection for the predictive model

We assessed different potential sets of predictor variables for input into the predictive model using the following criteria: (i) they allow prediction for at least half the genes in the genome, (ii) the Spearman correlation between all pairs of predictor variables is less than 0.3, (iii) they are drawn from different broad categories (genomic, evolutionary, functional and network) if possible, iv) achieve best performance in model assessment (see below).

### Assessment of model performance

The sensitivity of the prediction was plotted against (1 - specificity) and the area under the ROC curve (AUC) [44] was used as quantitative measure of the performance of the model, where sensitivity = 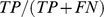, and specificity = 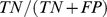. The other measure used is the Matthews correlation coefficients (MCC) [45], defined as:
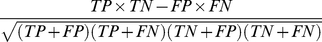
To avoid over-fitting, the sensitivity and specificity were calculated using 10-fold cross-validation. To overcome the variability caused by random partition involved in 10-fold cross-validation, each such assessment was repeated 30 times and the mean values were reported.

## Supporting Information

Dataset S1 HI_prediction.bed: Gene-based probability of exhibiting haploinsufficiency in BED format which can be loaded into UCSC genome browser.
(0.95 MB TXT)

Dataset S2 HI_prediction_with_imputation.bed: Gene-based probability of exhibiting haploinsufficiency (generated using gene properites that include imputated values) in BED format which can be loaded into UCSC genome browser.
(1.33 MB TXT)

Figure S1 Procedure for LOF calling. The flow chart shows the pipeline used to identify LOF genes. A gene with all its transcripts disrupted under any of the four considered LOF scenarios is regarded as LOF. On the right, the numbers under each scenario denotes the number of detected LOF events meeting that criteria. A LOF event is defined as loss of function of one transcript in one individual.
(0.24 MB PDF)

Figure S2 Number of human haplosufficient genes discovered from Affymetrix 6.0 array. The plot shows the number of LOF genes discovered as a function of the number of apparently healthy individuals being assayed. The red line represents all LOF genes whereas the gene line represents recurrent LOF genes, *i.e.* HS genes.
(0.08 MB PDF)

Figure S3 Comparison of model performance. The AUCs of each combination of predictor variables in 10-fold cross validation repeated 30 times are shown as vertical bars with error bars representing 2 times standard deviation. The mean AUC (red), mean MCC (green) and the overall gene coverage (blue) are labeled on top of each bar. The bar pointed to by the black arrowhead is the chosen combination of predictor variables.
(0.29 MB PDF)

Figure S4 Prediction performance of single predictor variable and integrated model. Mean AUC of each model in 10-fold cross-validation repeated 30 times are shown as vertical bars with the actual values label at the top.
(0.09 MB PDF)

Figure S5 Prediction performance of using HS and genome background as negative training. The plot compares the cross-validation performances resulted from using different gene sets as negative training set. The triangle represents HS gene set generated from CNV data. The squares represent different sizes of random gene sets sampled from the genome after excluding known HI genes. For each size, the gene set was sampled 20 times and the standard deviation of the resulting performances is shown as error bar.
(0.07 MB PDF)

Figure S6 Prediction performance under different parameters used in generation of negative training set. The cross-validation performance (AUC) resulted from using negative training sets generated with different parameters are represented by blue vertical bars with axis on the left. The sizes of these negative training sets are represented by red vertical bars with axis on the right. Bars are grouped by the CNV calling parameters, LOD score, and within each group the darkness of coloring represent different frequency threshold used to define HS as shown in the legend. The bar pointed by the black arrowhead represent parameters and corresponding negative training set adopted in further analysis.
(0.11 MB PDF)

Figure S7 Comparing the prediction performance of LDA and SVM. The plot shows the comparison of prediction performance between LDA (dark bar) and SVM (light bar) using three approaches (from left to right): self-validation, leave-one-out cross-validation and 10-fold cross-validation. In the first two comparisons, SVM exhibits only very marginal improvement over LDA, whereas in the third LDA is marginally better.
(0.01 MB PDF)

Figure S8 Examples of highlighting candidate genes, the 8p23.1 deletion. GATA4, the gene whose haploinsufficiency is attributed to the congenital heart malformation phenotype of the 8p23.1 deletion syndrome, is shown in this screenshot of the DECIPHER web browser to have the highest predicted haploinsufficiency of all 24 genes in this 3.4 Mb deletion interval.
(0.02 MB PNG)

Figure S9 Derived allele frequency spectrum of variants in different gene sets. This figure shows the spectrum of derived allele frequency (DAF, represented here as counts of derived allele in the population) of nonsynonymous SNPs and synonymous SNPs discovered by resequencing of human genes in a) 15 African Americans and b) 20 European Americans. In each plot, DAF of variants located in genes of different p(HI) are compared side by side, where bars of decreasing darkness represent quantiles of decreasing p(HI), such that the 0–25% quartile is that with the highest probability of being haploinsufficient.
(0.31 MB PDF)

Figure S10 Assessment of model performance after imputation. The ROC curve demonstrates the performance of the model trained on the enlarged training set using 10-fold cross-validation. The error bars represent standard errors of the mean. The lower right inset shows the relative contribution of each predictor variable to the prediction model measured by the absolute value of the scaling factor of each predictor variable constituting the linear discriminant.
(0.11 MB PDF)

Figure S11 Enrichment of predicted HI genes in dominant genes relative to recessive genes. The plot compares the fold of enrichment of predicted HI genes in dominant genes relative to recessive genes before (red line and circle) and after (blue line and triangle) imputation under a shifting threshold of p(HI) above which genes are regarded as HI.
(0.08 MB PDF)

Figure S12 Enrichment of predicted HI genes in orthologs of mouse haploinsufficient genes and mouse haplolethal genes. The plot compares the fold of enrichment of predicted HI genes in human orthologs of mouse haploinsufficient genes (red lines) and mouse haplolethal genes (blue lines) relative to the genome average before (darker lines with squares) and after (lighter lines with triangles) imputation under a shifting threshold of p(HI) above which genes are regarded as HI.
(0.08 MB PDF)

Protocol S1 Calculating LOD scores. Script and input files for calculating the LOD score of a deletion.
(3.04 MB ZIP)

Table S1 Composition of negative training set.
(0.03 MB PDF)

Table S2 Genomic coverage of gene properties.
(0.07 MB PDF)

Table S3 Spearman correlation between pairs of gene properties.
(0.07 MB PDF)

Table S4 Comparison of LOF deletions between European and African Americans.
(0.09 MB PDF)

Table S5 Number of genes with missing values in predictor variables.
(0.07 MB PDF)

Text S1 Supplementary notes.
(0.23 MB PDF)
