# Supplementary figures and images for: Characterising and Predicting Haploinsufficiency in the Human Genome
Source: PLoS Genet. 2010 Oct 14;6(10):e1001154. doi: 10.1371/journal.pgen.1001154 (PMC2954820; doi:10.1371/journal.pgen.1001154)

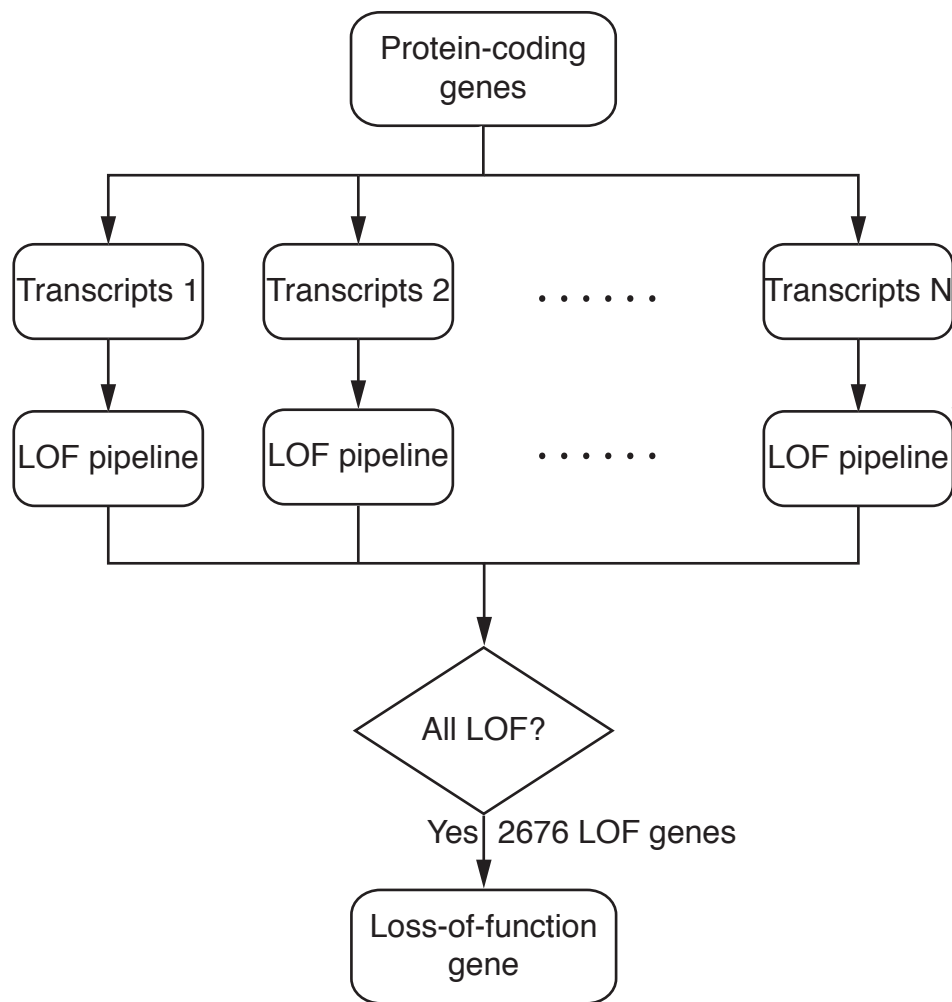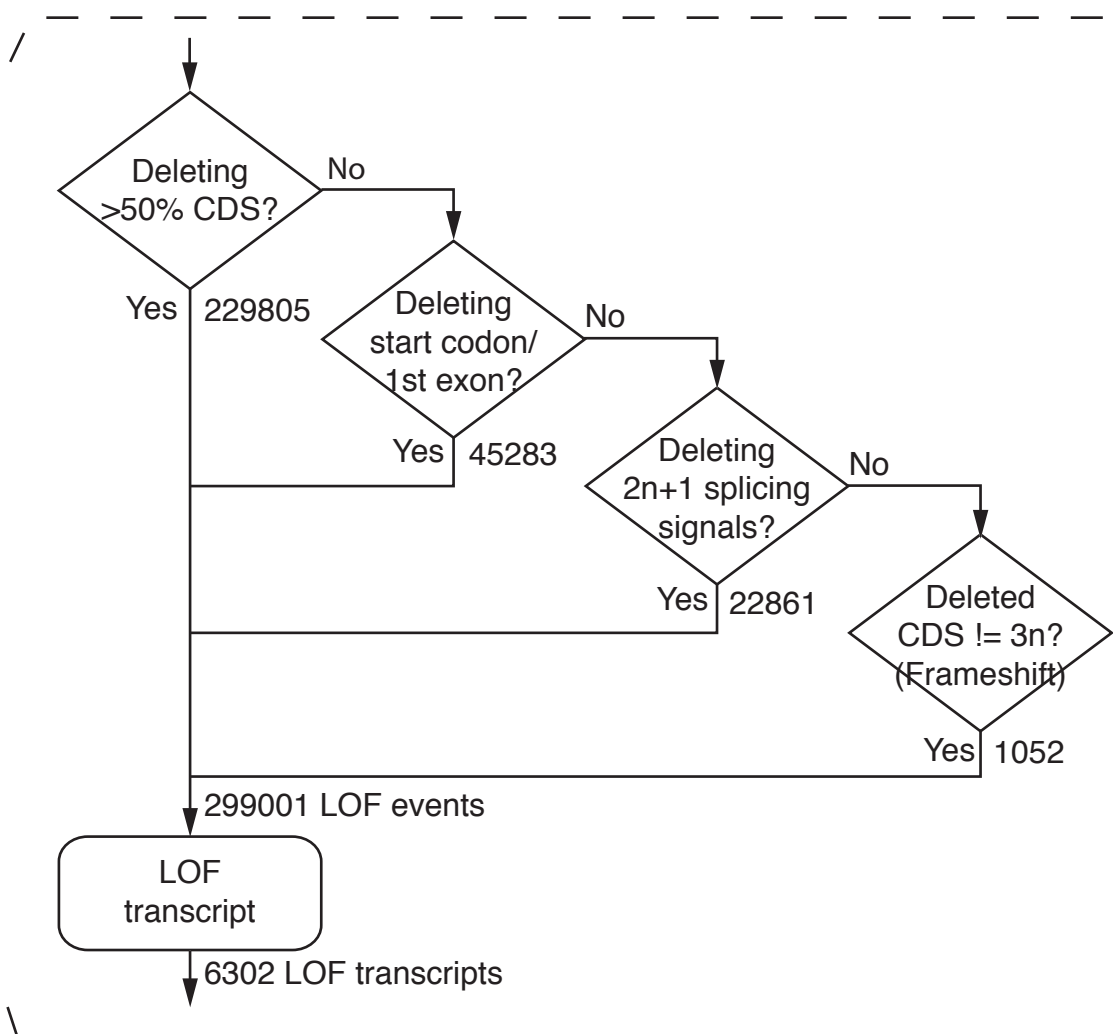

Supplement: Figure S1 — Procedure for LOF calling. The flow chart shows the pipeline used to identify LOF genes. A gene with all its transcripts disrupted under any of the four considered LOF scenarios is regarded as LOF. On the right, the numbers under each scenario denotes the number of detected LOF events meeting that criteria. A LOF event is defined as loss of function of one transcript in one individual. (0.24 MB PDF) [file pgen.1001154.s003.pdf]

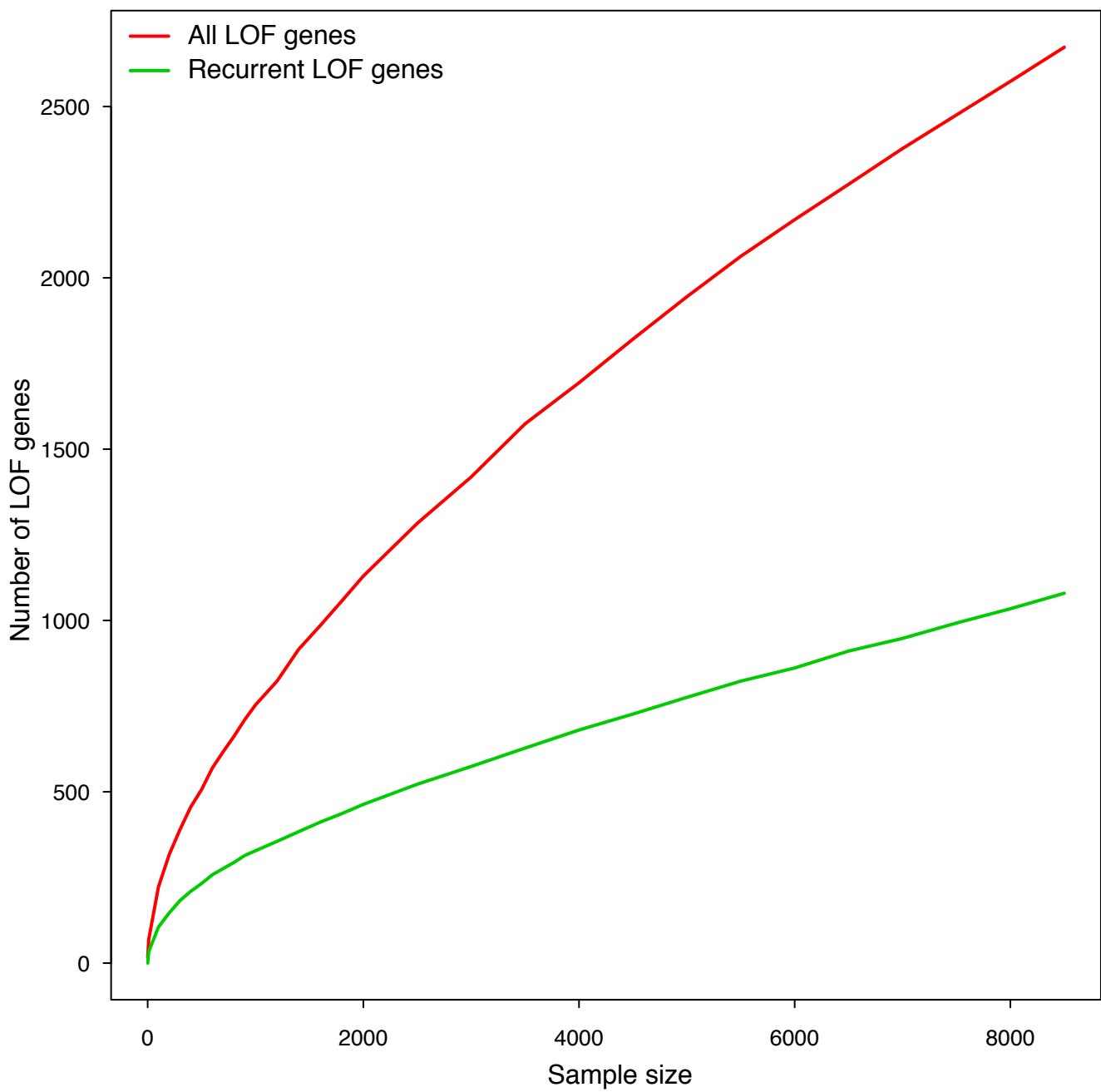

Supplement: Figure S2 — Number of human haplosufficient genes discovered from Affymetrix 6.0 array. The plot shows the number of LOF genes discovered as a function of the number of apparently healthy individuals being assayed. The red line represents all LOF genes whereas the gene line represents recurrent LOF genes, i.e. HS genes. (0.08 MB PDF) [file pgen.1001154.s004.pdf]

AUC

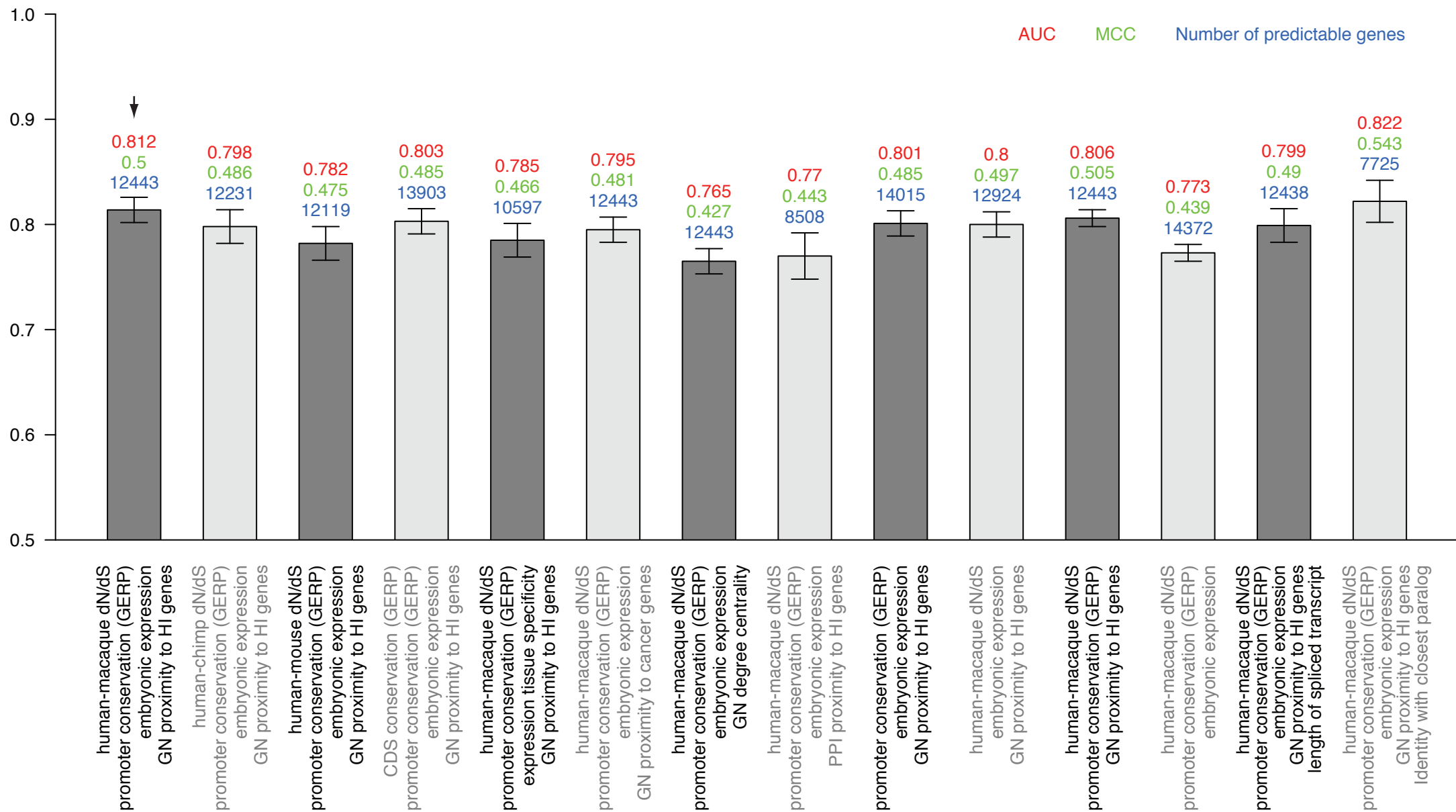

Supplement: Figure S3 — Comparison of model performance. The AUCs of each combination of predictor variables in 10-fold cross validation repeated 30 times are shown as vertical bars with error bars representing 2 times standard deviation. The mean AUC (red), mean MCC (green) and the overall gene coverage (blue) are labeled on top of each bar. The bar pointed to by the black arrowhead is the chosen combination of predictor variables. (0.29 MB PDF) [file pgen.1001154.s005.pdf]

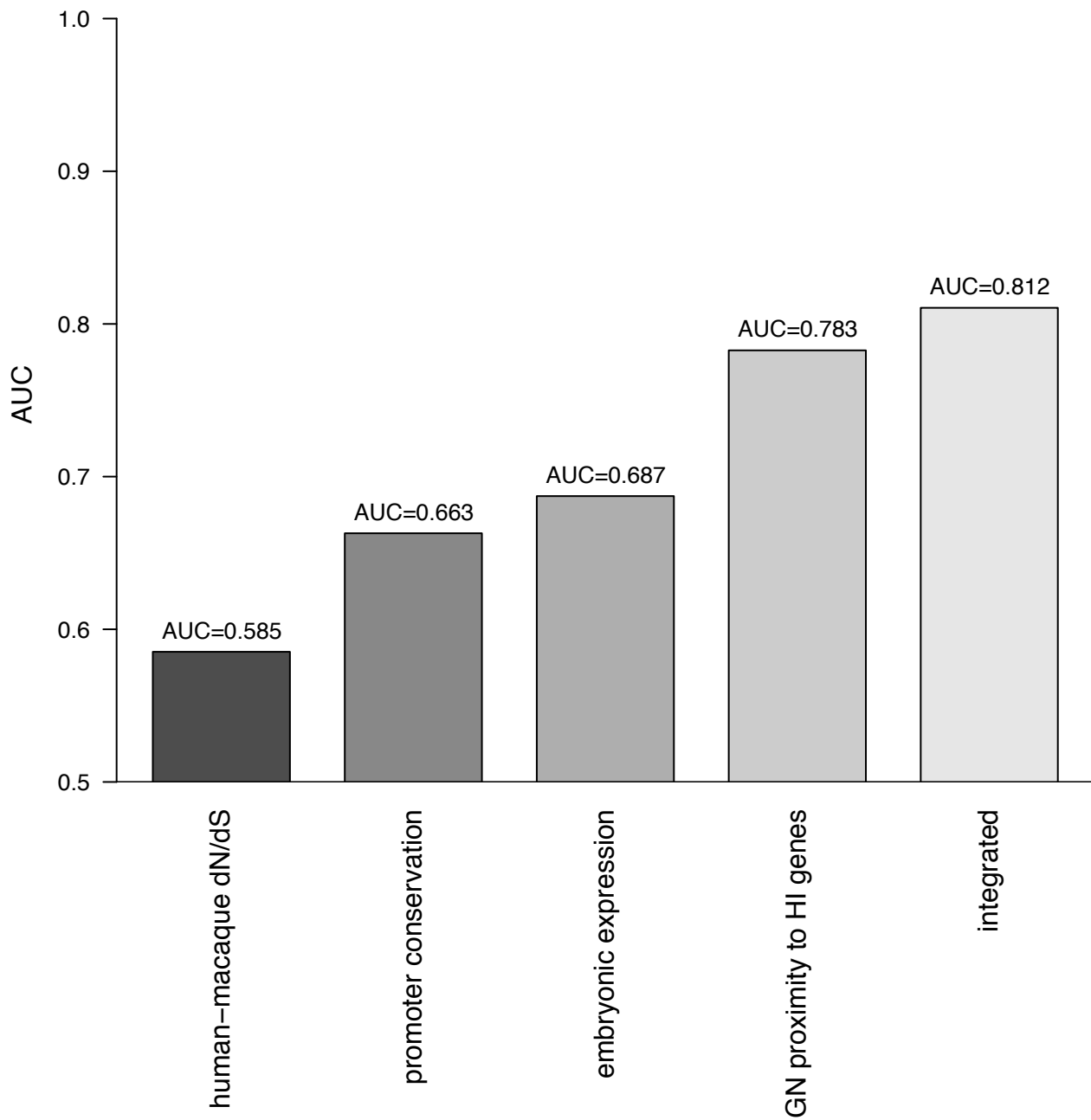

Supplement: Figure S4 — Prediction performance of single predictor variable and integrated model. Mean AUC of each model in 10-fold cross-validation repeated 30 times are shown as vertical bars with the actual values label at the top. (0.09 MB PDF) [file pgen.1001154.s006.pdf]

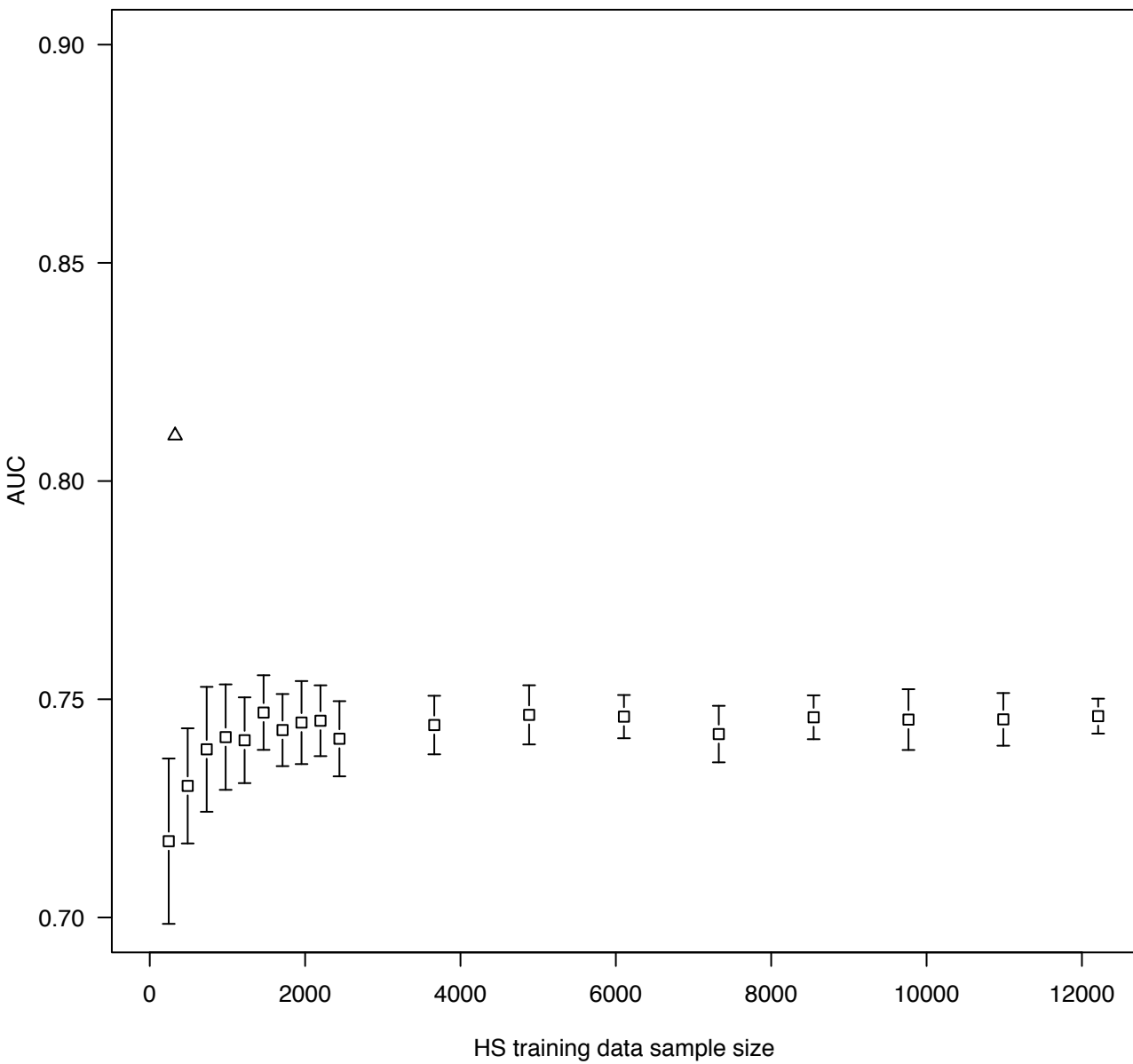

Supplement: Figure S5 — Prediction performance of using HS and genome background as negative training. The plot compares the cross-validation performances resulted from using different gene sets as negative training set. The triangle represents HS gene set generated from CNV data. The squares represent different sizes of random gene sets sampled from the genome after excluding known HI genes. For each size, the gene set was sampled 20 times and the standard deviation of the resulting performances is shown as error bar. (0.07 MB PDF) [file pgen.1001154.s007.pdf]

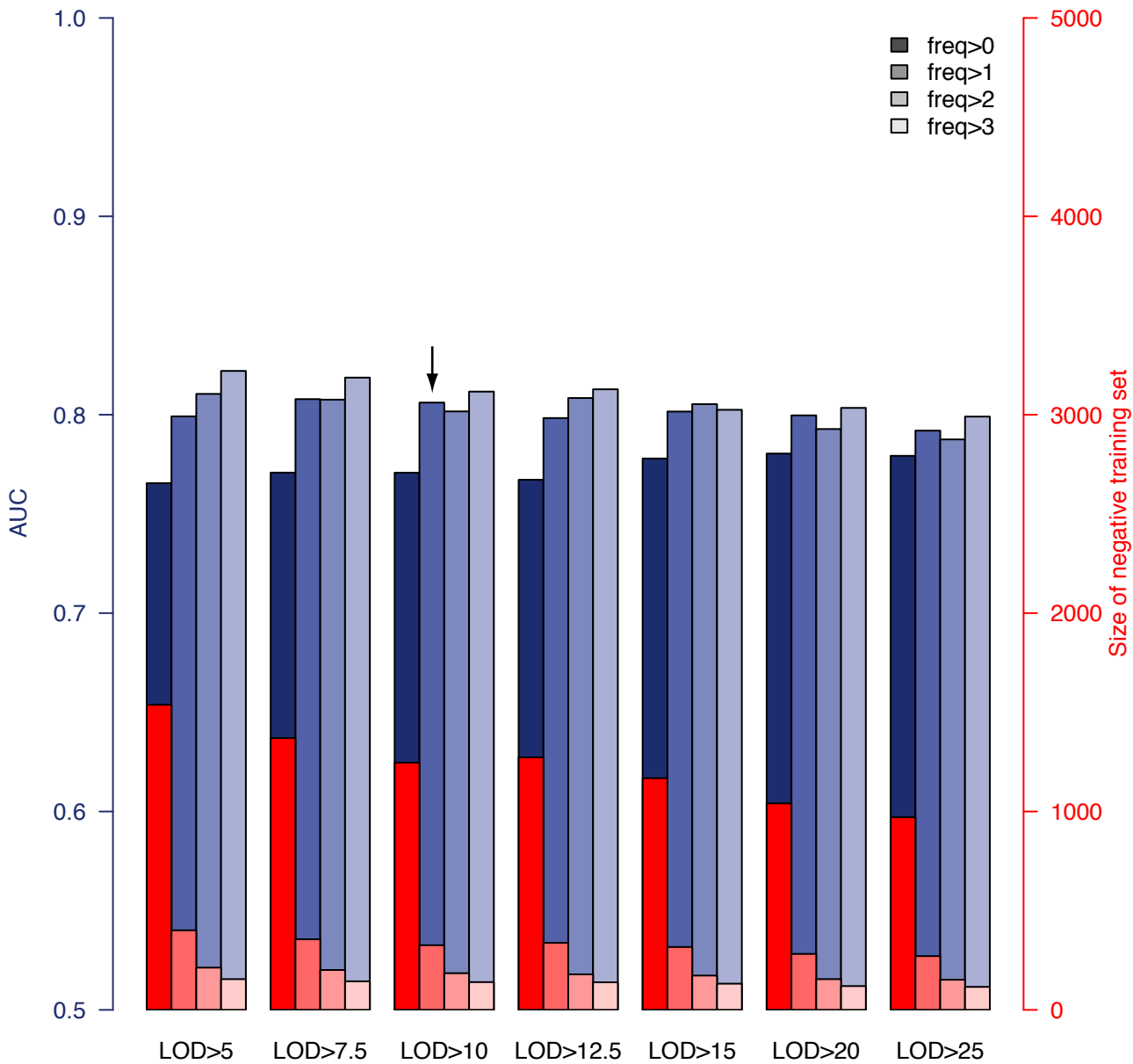

Supplement: Figure S6 — Prediction performance under different parameters used in generation of negative training set. The cross-validation performance (AUC) resulted from using negative training sets generated with different parameters are represented by blue vertical bars with axis on the left. The sizes of these negative training sets are represented by red vertical bars with axis on the right. Bars are grouped by the CNV calling parameters, LOD score, and within each group the darkness of coloring represent different frequency threshold used to define HS as shown in the legend. The bar pointed by the black arrowhead represent parameters and corresponding negative training set adopted in further analysis. (0.11 MB PDF) [file pgen.1001154.s008.pdf]

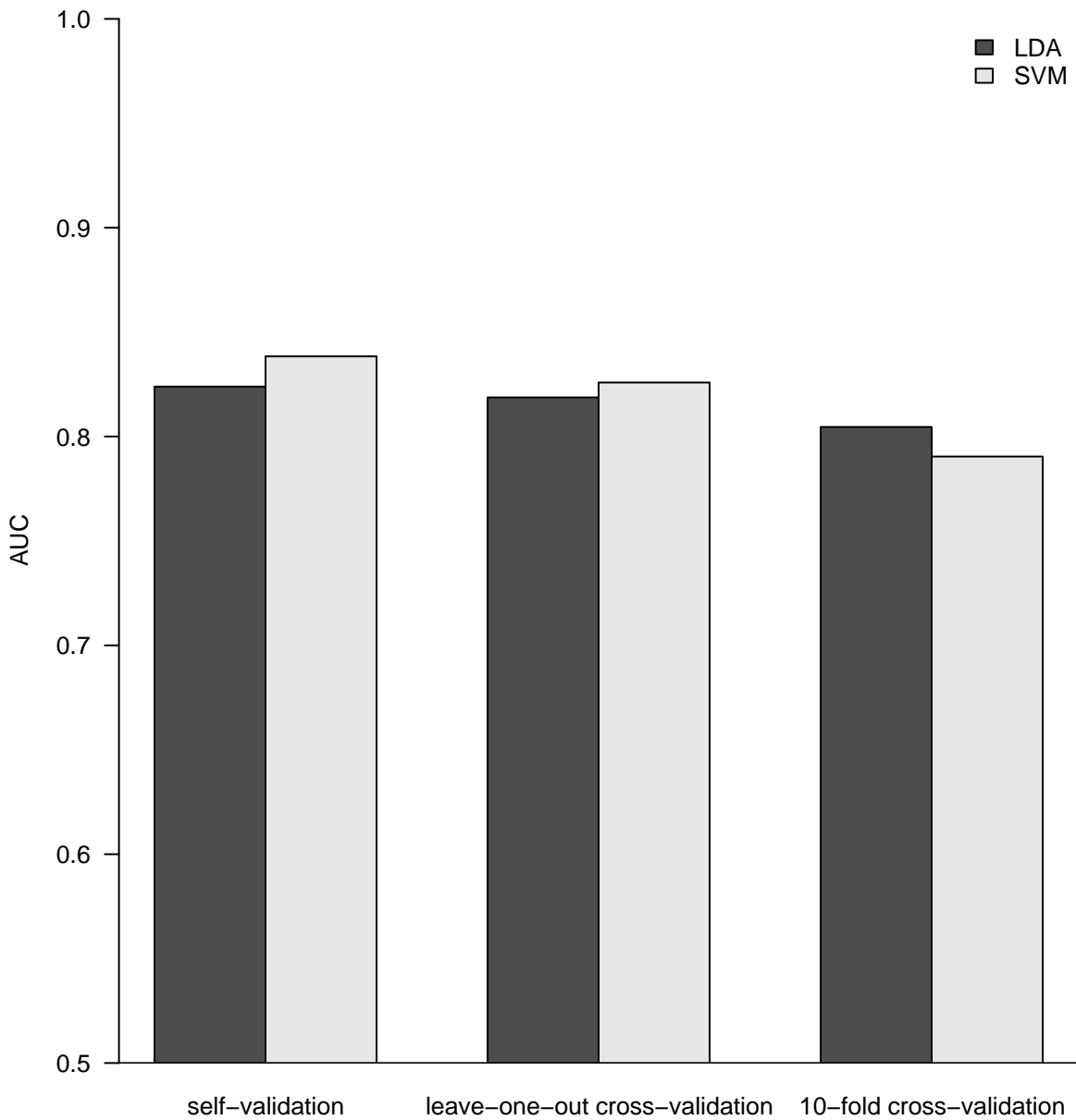

Supplement: Figure S7 — Comparing the prediction performance of LDA and SVM. The plot shows the comparison of prediction performance between LDA (dark bar) and SVM (light bar) using three approaches (from left to right): self-validation, leave-one-out cross-validation and 10-fold cross-validation. In the first two comparisons, SVM exhibits only very marginal improvement over LDA, whereas in the third LDA is marginally better. (0.01 MB PDF) [file pgen.1001154.s009.pdf]

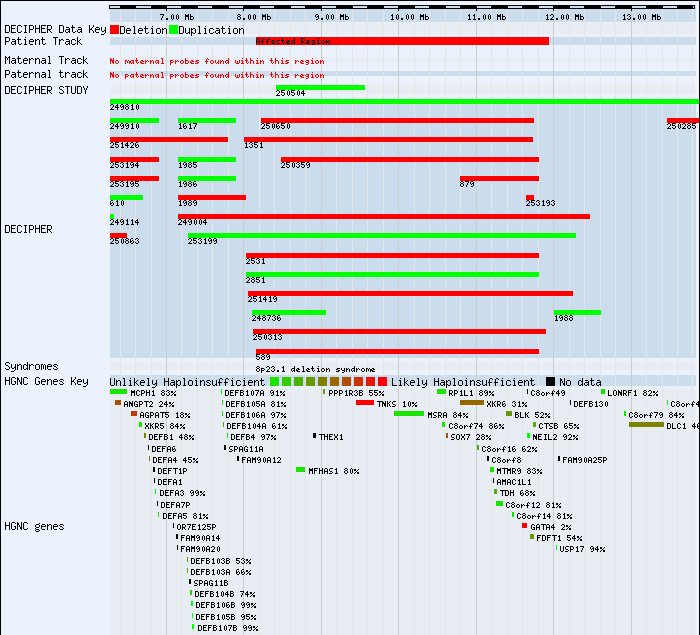

Supplement: Figure S8 — Examples of highlighting candidate genes, the 8p23.1 deletion. GATA4, the gene whose haploinsufficiency is attributed to the congenital heart malformation phenotype of the 8p23.1 deletion syndrome, is shown in this screenshot of the DECIPHER web browser to have the highest predicted haploinsufficiency of all 24 genes in this 3.4 Mb deletion interval. (0.02 MB PNG) [file pgen.1001154.s010.png]

a

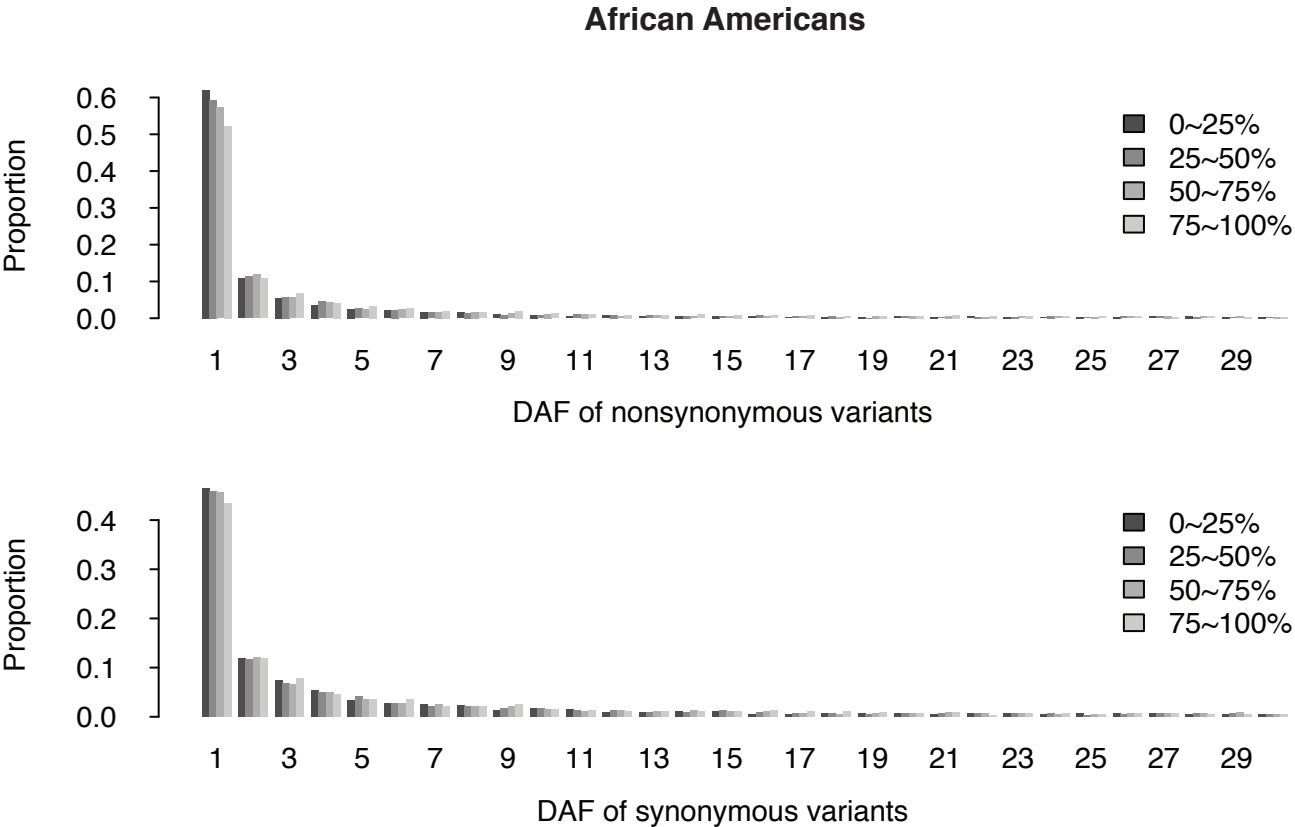

b

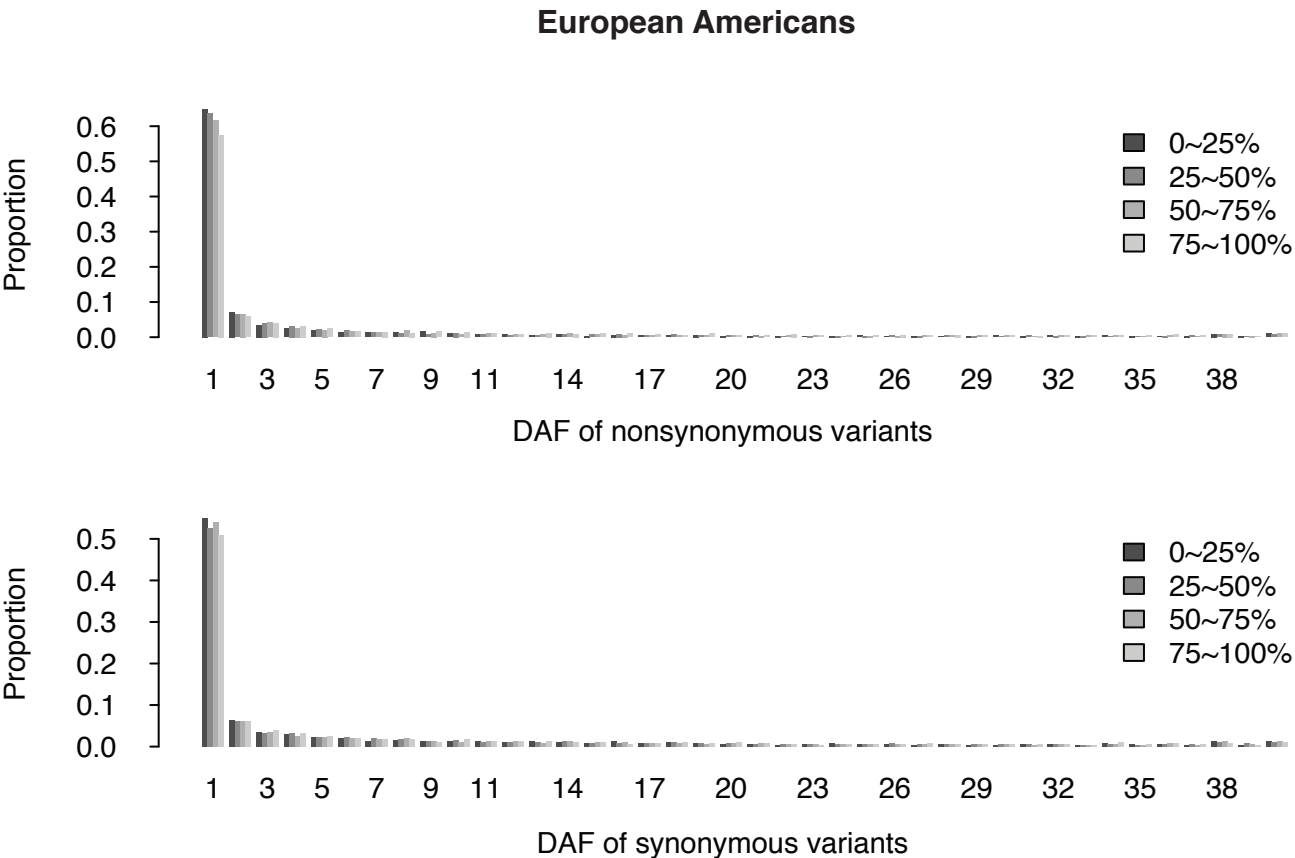

Supplement: Figure S9 — Derived allele frequency spectrum of variants in different gene sets. This figure shows the spectrum of derived allele frequency (DAF, represented here as counts of derived allele in the population) of nonsynonymous SNPs and synonymous SNPs discovered by resequencing of human genes in a) 15 African Americans and b) 20 European Americans. In each plot, DAF of variants located in genes of different p(HI) are compared side by side, where bars of decreasing darkness represent quantiles of decreasing p(HI), such that the 0–25% quartile is that with the highest probability of being haploinsufficient. (0.31 MB PDF) [file pgen.1001154.s011.pdf]

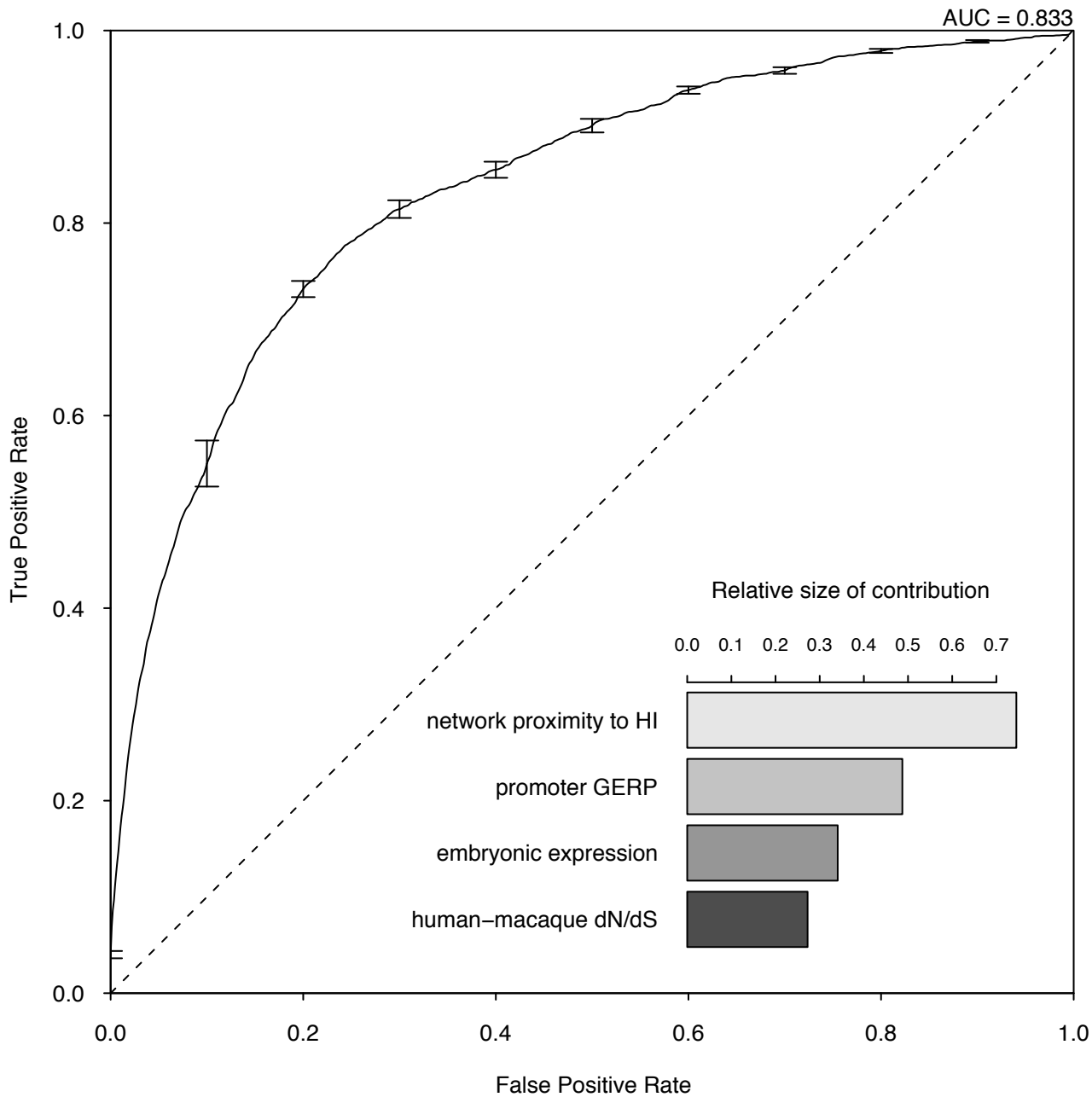

Supplement: Figure S10 — Assessment of model performance after imputation. The ROC curve demonstrates the performance of the model trained on the enlarged training set using 10-fold cross-validation. The error bars represent standard errors of the mean. The lower right inset shows the relative contribution of each predictor variable to the prediction model measured by the absolute value of the scaling factor of each predictor variable constituting the linear discriminant. (0.11 MB PDF) [file pgen.1001154.s012.pdf]

Fold of enrichment

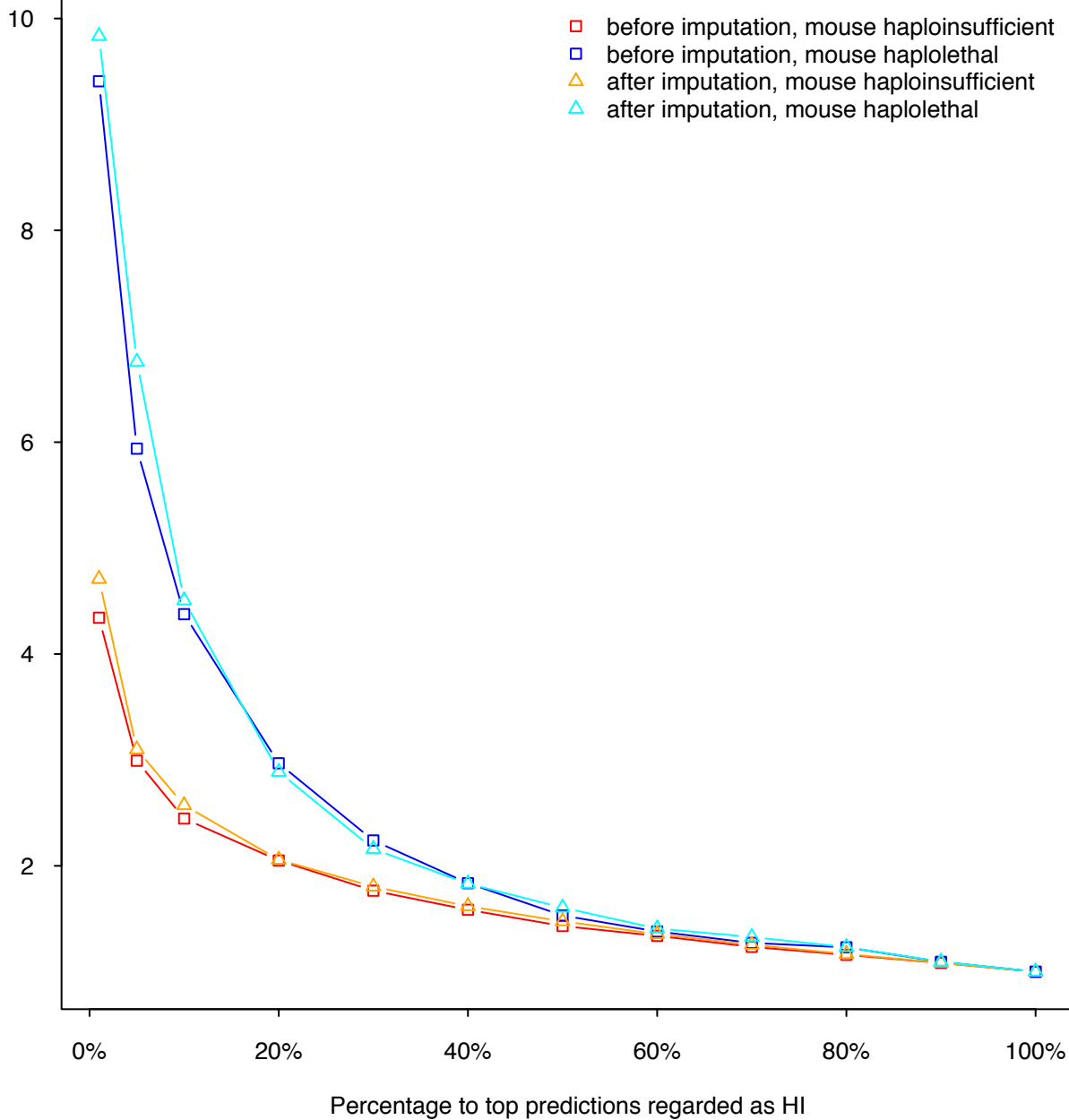

Supplement: Figure S12 — Enrichment of predicted HI genes in orthologs of mouse haploinsufficient genes and mouse haplolethal genes. The plot compares the fold of enrichment of predicted HI genes in human orthologs of mouse haploinsufficient genes (red lines) and mouse haplolethal genes (blue lines) relative to the genome average before (darker lines with squares) and after (lighter lines with triangles) imputation under a shifting threshold of p(HI) above which genes are regarded as HI. (0.08 MB PDF) [file pgen.1001154.s014.pdf]
