# Supplementary material for: Characterising and Predicting Haploinsufficiency in the Human Genome
Source: PLoS Genet. 2010 Oct 14;6(10):e1001154. doi: 10.1371/journal.pgen.1001154 (PMC2954820; doi:10.1371/journal.pgen.1001154)
Supplement: Figure S11 — Enrichment of predicted HI genes in dominant genes relative to recessive genes. The plot compares the fold of enrichment of predicted HI genes in dominant genes relative to recessive genes before (red line and circle) and after (blue line and triangle) imputation under a shifting threshold of p(HI) above which genes are regarded as HI. (0.08 MB PDF) [file pgen.1001154.s013.pdf]

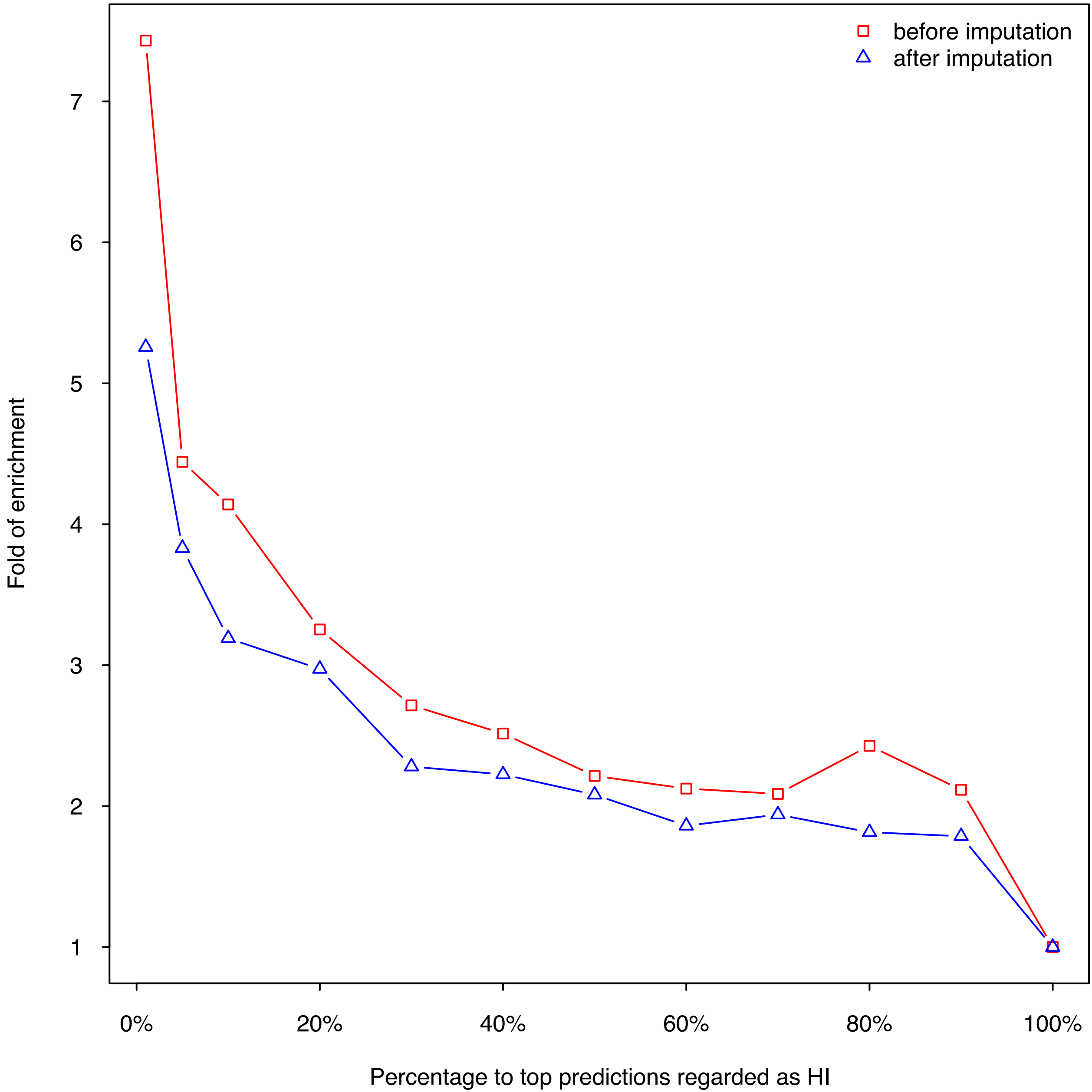

before imputation
after imputation
7
6
5
4
3
2
1
Fold of enrichment
0%
20%
40%
60%
80%
100%
Percentage to top predictions regarded as HI
