# Supplementary material for: Characterising and Predicting Haploinsufficiency in the Human Genome
Source: PLoS Genet. 2010 Oct 14;6(10):e1001154. doi: 10.1371/journal.pgen.1001154 (PMC2954820; doi:10.1371/journal.pgen.1001154)
Supplement: Table S1 — Composition of negative training set. (0.03 MB PDF) [file pgen.1001154.s016.pdf]

Table S1: **Composition of negative training set**

Source	Sample size	CNV calls	LOF events	Remove >50% CDS	Remove start codon or the first exon	Remove splicing signals	Cause frame-shift	LOF transcript	LOF genes	Full LOF genes	Recurrent LOF genes
WTCCC2	5,929	333,744	213,386	162,251	33,764	16,624	747	4,651	2,401	1,977	
GAIN	2,322	112,505	78,297	61,872	10,392	5,748	285	2,973	1,607	1,342	
HapMap	207	11,722	7,318	5,682	1,127	489	20	801	447	375	
Total	8,458	457,971	299,001	229,805	45,283	22,861	1,052	6,302	3,230	2,676	1,079
