# Supplementary material for: Characterising and Predicting Haploinsufficiency in the Human Genome
Source: PLoS Genet. 2010 Oct 14;6(10):e1001154. doi: 10.1371/journal.pgen.1001154 (PMC2954820; doi:10.1371/journal.pgen.1001154)
Supplement: Table S2 — Genomic coverage of gene properties. (0.07 MB PDF) [file pgen.1001154.s017.pdf]

Table S2: **Genomic coverage of gene properties**

Property	Number of genes	Genomic coverage*
dN/dS human-chimp	15,084	79.5%
dN/dS human-macaque	15,025	79.2%
dN/dS human-mouse	14,386	75.8%
Coding seqeunce GERP	17,164	90.5%
Promoter GERP	16,807	88.7%
Number of paralogs<br>Identity of closest paralogs	11,066	58.3%
Number of exons<br>Length of gene<br>Length of spliced transcript<br>Length of coding sequence<br>Length of 3'UTR	17,700	93.3%
Number of domains	14,722	88.5%
Embryonic expression†	14,722 (2,421)	88.5% (12.8%)
Tissue specificity of expression	13,950	73.6%
PPI network properties‡	11,077	58.4%
Genetic network properties‡	14,664	77.3%
+/- yeast growth rate	3,352	17.7%

* Calculated relative to the number of EnsEMBL annotated protein-coding genes that can be uniquely mapped to HGNC symbol.

† Since this is a binary factor where every gene is classified as either over-expressed or not in embryo tissue, the coverage is 100%. The number and proportion of genes over-expressed in embryo is listed in parenthesis.

‡ Including degree, cluster coefficient, betweenness, distance to known HI genes/cancer genes, proximity to known HI/cancer genes.
