# Supplementary material for: Characterising and Predicting Haploinsufficiency in the Human Genome
Source: PLoS Genet. 2010 Oct 14;6(10):e1001154. doi: 10.1371/journal.pgen.1001154 (PMC2954820; doi:10.1371/journal.pgen.1001154)
Supplement: Table S3 — Spearman correlation between pairs of gene properties. (0.07 MB PDF) [file pgen.1001154.s018.pdf]

## Table S3: Spearman correlation between pairs of gene properties

| | human-chimp $dN/dS$ 1 | human-macaque $dN/dS$ 2 | human-mouse $dN/dS$ 3 | CDS conservation (GERP) 4 | promoter conservation (GERP) 5 | number of paralogs 6 | identity to closest paralog 7 | number of exons 8 | length of gene 9 | length of spliced transcript 10 | length of CDS 11 | length of 3'UTR 12 | number of protein domains 13 | embryonic expression 14 | expression tissue specificity 15 | PPI degree centrality* 16 | PPI cluster coefficient 17 | PPI betweenness centrality 18 | PPI distance to HI genes 19 | PPI proximity to HI genes 20 | PPI distance to cancer genes 21 | PPI proximity to cancer genes 22 | GN degree centrality† 23 | GN cluster coefficient 24 | GN betweenness centrality 25 | GN distance to HI genes 26 | GN proximity to HI genes 27 | GN distance to cancer genes 28 | GN proximity to cancer genes 29 | +/- yeast growth rate 30 |
|---|---|---|---|---|---|---|---|---|---|---|---|---|---|---|---|---|---|---|---|---|---|---|---|---|---|---|---|---|---|---|
| **1** | 1.00 | | | | | | | | | | | | | | | | | | | | | | | | | | | | | |
| **2** | 0.55 | 1.00 | | | | | | | | | | | | | | | | | | | | | | | | | | | | |
| **3** | 0.55 | 0.69 | 1.00 | | | | | | | | | | | | | | | | | | | | | | | | | | | |
| **4** | -0.39 | -0.41 | -0.51 | 1.00 | | | | | | | | | | | | | | | | | | | | | | | | | | |
| **5** | -0.20 | -0.23 | -0.27 | 0.40 | 1.00 | | | | | | | | | | | | | | | | | | | | | | | | | |
| **6** | 0.06 | 0.05 | 0.01 | -0.13 | -0.07 | 1.00 | | | | | | | | | | | | | | | | | | | | | | | | |
| **7** | 0.06 | 0.05 | 0.01 | -0.13 | -0.07 | 1.00 | 1.00 | | | | | | | | | | | | | | | | | | | | | | | |
| **8** | -0.06 | -0.03 | -0.09 | 0.26 | 0.14 | -0.14 | -0.13 | 1.00 | | | | | | | | | | | | | | | | | | | | | | |
| **9** | -0.10 | -0.07 | -0.12 | 0.32 | 0.22 | -0.14 | -0.13 | 0.66 | 1.00 | | | | | | | | | | | | | | | | | | | | | |
| **10** | -0.09 | -0.13 | -0.15 | 0.21 | 0.24 | -0.09 | -0.13 | 0.55 | 0.58 | 1.00 | | | | | | | | | | | | | | | | | | | | |
| **11** | 0.02 | -0.02 | -0.04 | 0.09 | 0.17 | -0.05 | -0.13 | 0.67 | 0.51 | 0.74 | 1.00 | | | | | | | | | | | | | | | | | | | |
| **12** | -0.12 | -0.16 | -0.16 | 0.21 | 0.20 | -0.05 | -0.02 | 0.11 | 0.35 | 0.63 | 0.15 | 1.00 | | | | | | | | | | | | | | | | | | |
| **13** | -0.05 | -0.06 | -0.11 | 0.03 | 0.09 | 0.02 | 0.03 | 0.36 | 0.28 | 0.35 | 0.43 | 0.09 | 1.00 | | | | | | | | | | | | | | | | | |
| **14** | -0.05 | -0.04 | -0.04 | 0.12 | 0.06 | 0.01 | 0.01 | 0.10 | 0.08 | 0.08 | 0.08 | 0.05 | 0.05 | 1.00 | | | | | | | | | | | | | | | | |
| **15** | -0.09 | -0.05 | -0.04 | 0.09 | 0.04 | 0.00 | 0.00 | 0.08 | 0.00 | -0.04 | -0.08 | -0.05 | 0.00 | 0.20 | 1.00 | | | | | | | | | | | | | | | |
| **16** | -0.15 | -0.14 | -0.19 | 0.17 | 0.07 | 0.02 | 0.12 | 0.09 | 0.03 | 0.03 | 0.02 | 0.01 | 0.12 | 0.09 | 0.12 | 1.00 | | | | | | | | | | | | | | |
| **17** | -0.08 | -0.08 | -0.10 | 0.11 | 0.04 | 0.00 | 0.05 | 0.03 | 0.00 | 0.00 | 0.00 | 0.00 | 0.04 | 0.06 | 0.03 | 0.62 | 1.00 | | | | | | | | | | | | | |
| **18** | -0.09 | -0.08 | -0.13 | 0.11 | 0.06 | 0.01 | 0.07 | 0.08 | 0.03 | 0.03 | 0.03 | 0.02 | 0.09 | 0.07 | 0.09 | 0.63 | 0.29 | 1.00 | | | | | | | | | | | | |
| **19** | 0.09 | 0.09 | 0.10 | -0.09 | -0.06 | 0.01 | -0.08 | -0.06 | -0.02 | -0.02 | 0.00 | -0.01 | -0.07 | -0.06 | -0.08 | -0.55 | -0.34 | -0.38 | 1.00 | | | | | | | | | | | |
| **20** | -0.10 | -0.09 | -0.12 | 0.08 | 0.08 | 0.04 | 0.10 | 0.06 | 0.03 | 0.05 | 0.05 | 0.02 | 0.13 | 0.08 | 0.08 | 0.62 | 0.36 | 0.42 | -0.57 | 1.00 | | | | | | | | | | |
| **21** | 0.09 | 0.08 | 0.10 | -0.11 | -0.07 | 0.01 | -0.06 | -0.06 | -0.02 | -0.02 | 0.00 | -0.01 | -0.06 | -0.07 | -0.07 | -0.55 | -0.35 | -0.37 | 0.82 | -0.44 | 1.00 | | | | | | | | | |
| **22** | -0.11 | -0.11 | -0.14 | 0.14 | 0.08 | 0.00 | 0.07 | 0.06 | 0.02 | 0.05 | 0.03 | 0.03 | 0.11 | 0.08 | 0.05 | 0.60 | 0.38 | 0.39 | -0.44 | 0.62 | -0.55 | 1.00 | | | | | | | | |
| **23** | -0.17 | -0.16 | -0.22 | 0.23 | 0.07 | -0.03 | 0.14 | 0.18 | 0.07 | 0.05 | 0.05 | 0.00 | 0.14 | 0.14 | 0.24 | 0.49 | 0.29 | 0.29 | -0.29 | 0.32 | -0.30 | 0.37 | 1.00 | | | | | | | |
| **24** | 0.05 | 0.05 | 0.07 | -0.07 | -0.07 | 0.01 | 0.00 | -0.08 | -0.08 | -0.10 | -0.07 | -0.08 | -0.05 | -0.02 | -0.03 | -0.08 | 0.11 | -0.12 | 0.06 | -0.11 | 0.06 | -0.06 | 0.15 | 1.00 | | | | | | |
| **25** | -0.17 | -0.16 | -0.21 | 0.21 | 0.09 | -0.01 | 0.12 | 0.18 | 0.09 | 0.08 | 0.07 | 0.03 | 0.15 | 0.14 | 0.27 | 0.44 | 0.21 | 0.30 | -0.27 | 0.34 | -0.27 | 0.33 | 0.86 | -0.07 | 1.00 | | | | | |
| **26** | 0.11 | 0.12 | 0.15 | -0.15 | -0.06 | 0.01 | -0.08 | -0.10 | -0.04 | -0.05 | -0.04 | -0.01 | -0.09 | -0.10 | -0.13 | -0.25 | -0.15 | -0.16 | 0.23 | -0.24 | 0.23 | -0.23 | -0.51 | -0.05 | -0.47 | 1.00 | | | | |
| **27** | -0.14 | -0.17 | -0.21 | 0.19 | 0.09 | -0.02 | 0.12 | 0.12 | 0.06 | 0.09 | 0.09 | 0.03 | 0.12 | 0.14 | 0.13 | 0.39 | 0.22 | 0.24 | -0.29 | 0.41 | -0.28 | 0.38 | 0.66 | -0.01 | 0.60 | -0.57 | 1.00 | | | |
| **28** | 0.11 | 0.10 | 0.14 | -0.14 | -0.08 | 0.02 | -0.08 | -0.11 | -0.06 | -0.06 | -0.05 | -0.03 | -0.10 | -0.10 | -0.14 | -0.29 | -0.17 | -0.19 | 0.25 | -0.26 | 0.34 | -0.34 | -0.54 | -0.06 | -0.49 | 0.75 | -0.44 | 1.00 | | |
| **29** | -0.15 | -0.16 | -0.21 | 0.20 | 0.11 | -0.03 | 0.12 | 0.15 | 0.07 | 0.10 | 0.08 | 0.05 | 0.14 | 0.14 | 0.15 | 0.47 | 0.30 | 0.29 | -0.32 | 0.40 | -0.38 | 0.59 | 0.72 | 0.02 | 0.63 | -0.43 | 0.67 | -0.60 | 1.00 | |
| **30** | -0.02 | 0.01 | 0.02 | 0.04 | 0.03 | 0.01 | -0.05 | 0.05 | 0.07 | 0.03 | -0.01 | 0.07 | 0.01 | -0.02 | 0.02 | -0.11 | -0.12 | -0.04 | 0.05 | -0.06 | 0.04 | -0.12 | -0.04 | -0.08 | -0.01 | 0.02 | -0.06 | 0.00 | -0.06 | 1.00 |

*PPI: protein-protein interaction network; †GN: genetic network
