# Supplementary material for: Characterising and Predicting Haploinsufficiency in the Human Genome
Source: PLoS Genet. 2010 Oct 14;6(10):e1001154. doi: 10.1371/journal.pgen.1001154 (PMC2954820; doi:10.1371/journal.pgen.1001154)
Supplement: Table S4 — Comparison of LOF deletions between European and African Americans. (0.09 MB PDF) [file pgen.1001154.s019.pdf]

Table S4: **Comparison of LOF deletions between European and African Americans**

Population	Average number of LOF CNVs per individual	Average number of predictable LOF genes per individual	Average number of LOF genes in most pathogenic CNV per individual*	Average LOD_{max} per individual	Proportion of population with LOD_{max} in the top 1% of the pooled population
African American	7.35	10.1	2.74	-0.38	0.79%
European American	7.41	10.5	2.85	-0.36	1.18%

* Most pathogenic defined as having the highest LOD score in the individual (LOD_{max}).
