# Supplementary material for: Characterising and Predicting Haploinsufficiency in the Human Genome
Source: PLoS Genet. 2010 Oct 14;6(10):e1001154. doi: 10.1371/journal.pgen.1001154 (PMC2954820; doi:10.1371/journal.pgen.1001154)
Supplement: Table S5 — Number of genes with missing values in predictor variables. (0.07 MB PDF) [file pgen.1001154.s020.pdf]

Table S5: **Number of genes with missing values in predictor variables**

dN/dS between human and macaque	Promoter conservation (GERP)	Embryonic expression	Proximity to HI genes in genetic network	Number of genes
$+$	$+$	$+$	$+$	12,443
$-$	$+$	$+$	$+$	1,572
$+$	$-$	$+$	$+$	481
$+$	$+$	$+$	$-$	1,929
$-$	$-$	$+$	$+$	168
$-$	$+$	$+$	$-$	863
$+$	$-$	$+$	$-$	172
$-$	$-$	$+$	$-$	1,334

$^{+}$ value available; $^{-}$ value missing
