# Supplementary material for: Characterising and Predicting Haploinsufficiency in the Human Genome
Source: PLoS Genet. 2010 Oct 14;6(10):e1001154. doi: 10.1371/journal.pgen.1001154 (PMC2954820; doi:10.1371/journal.pgen.1001154)
Supplement: Text S1 — Supplementary notes. (0.23 MB PDF) [file pgen.1001154.s021.pdf]

Supplementary notes to "Characterising and predicting haploinsufficiency in the human genome"

Ni Huang[1], Insuk Lee[2,3], Edward M. Marcotte[2], Matthew E. Hurles[1]

1. Wellcome Trust Sanger Institute, Wellcome Trust Genome Campus, Cambridge, UK

2. Center for Systems and Synthetic Biology, Department of Chemistry and Biochemistry, Institute for Cellular and Molecular Biology, University of Texas, 2500 Speedway, MBB 3.210, Austin, Texas 78712, USA

3. Department of Biotechnology, College of Life Science and Biotechnology, Yonsei University, 262 Seongsanno, Seodaemun-gu, Seoul 120-749, South Korea

Corresponding author:
Matthew Hurles
Wellcome Trust Sanger Institute,
Wellcome Trust Genome Campus,
Hinxton, Cambridge, UK, CB10 1SA
Tel: +44 (0)1223 495377
Fax: +44 (0)1223 494919
Email: meh@sanger.ac.uk

Contents

1 Identification of haploinsufficient genes from copy number variation data 3

1.1 CNV discovery . . . 3

1.2 Assertion of LOF . . . 3

1.3 Genomic coverage of the current map of haplosufficient genes . . . 3

2 Select predictor variables from gene properties 4

3 Assessment of model performance 4

3.1 Single predictor variable vs. integrated model . . . 4

3.2 Comparing the use of HS and genome background as negative training set . . . 4

3.3 Influence of CNV discovery parameters on prediction performance . . . 5

3.4 Comparing the use of LDA and SVM as classifier . . . 5

4 Validation using external data 5

4.1 Validation using genes implicated in genetic diseases with known mode of inheritance . . . 5

4.2 Validation using orthologs of mouse HI genes . . . 6

4.3 Validation using putative pathogenic deletions . . . 6

4.4 Allele frequency spectrum of nonsynonymous variants in genes with different p(HI) . . . 7

5 Expanding genomic coverage through imputation 7

5.1 Pattern of missing data . . . 7

5.2 Multiple imputation . . . 8

5.3 Model training and performance . . . 8

5.4 Validation using external data . . . 8

References 10

Figure legends 11

1 Identification of haploinsufficient genes from copy number variation data

1.1 CNV discovery

CNVs were discovered from three different sources of genotyping dataset generated on Affymetrix 6.0 platform, including 210 unrelated HapMap individuals [1], 6,000 UK healthy individuals used as controls for the WTCCC2 GWAS study and 2,421 healthy individuals used as controls in the GAIN GWAS study of Schizophrenia and Bipolar Disorders [2]. The three datasets were processed separately using the same procedure. For each dataset, original CEL files were run through Birdsuite [3] plate by plate using the default parameters and autosomal CNV calls with LOD score over 10 were kept and subjected to further filtering, which removed CNV calls that were smaller than one kilobase, or were supported by fewer than 5 probes, or had fewer than 1 probe per 10kb on average. The number of CNVs per individual and the total size of CNV affected region was then fitted to a normal distribution and apparently outlying individuals that gave over 100 CNV calls or over 10Mb of CNV affected region were removed. This resulted in 458,340 CNV calls from 8,458 apparently healthy individuals (Table S1).

1.2 Assertion of LOF

To identify protein-coding genes disrupted in a LOF manner, CNV calls were compared to gene annotation provided by EnsEMBL release 50 [4]. Four scenarios were considered LOF to a protein-coding transcript (Figure S1):

- deletion of over 50% of coding sequence
- deletion of the start codon or the first exon
- deletion-disrupted-splicing
- deletion-caused frame-shift

A gene was considered LOF if all of its transcripts were LOF. Under this criteria, 2,677 LOF genes were identified. We defined haplosufficient genes as being those observed as LOF genes in 2 or more individuals. The numbers of LOF transcripts and genes contributed from each of the three datasets are given in the Table S1.

1.3 Genomic coverage of the current map of haplosufficient genes

To investigate the coverage of the map of haplosufficient genes derived from the identification of LOF genes in apparently healthy individuals, we examined the number of LOF genes and haplosufficient genes identified as a function of the number of samples assayed on Affymetrix 6.0 (Figure S2). The increase

in the number of identified HS genes with the increase of the number of assayed individuals slows down dramatically as sample size increases beyond a few hundred. However, there is no sign of reaching an asympotic state in the number of HS genes, as a result of increasing numbers of low frequency CNVs being found with larger sample sizes.

2 Select predictor variables from gene properties

We aim to select a subset of relatively uncorrelated gene properties drawn from different classes of gene annotation as predictor variables to achieve best prediction performance for over half of the genome. To do this, we first calculated the Spearman correlation between each pair of gene properties (Table S3). We avoided selecting pairs with correlation above the threshold of $R^2 = 0.05$ into the model. We also examined the genomic coverage (Table S2) of combinations of predictor variables and excluded combinations that are available to less than half of the genome. In the end we chose the combination of predictor variables that achieved best performance in the assessment, although many alternate models had very similar performance (Figure S3). The final model consisted of four predictor variables:

- dN/dS ratio between human and macaque
- promoter conservation (GERP score)
- embryonic expression
- gene network proximity to HI genes

Note that although the model with 'identity with closest paralog' exhibited better performance (the rightmost bar in Figure S3), it failed our criteria of being available to over half of the genome and therefore was not incorporated.

3 Assessment of model performance

3.1 Single predictor variable vs. integrated model

To demonstrate the value of data integration in prediction, we trained separate LDA models from the same set of genes (known HI genes plus HS genes) using only one predictor variable at a time and compared the cross-validation performance with using all predictor variables (Figure S4).

3.2 Comparing the use of HS and genome background as negative training set

Previous studies [5–7] have compared HI-related gene sets against the rest of the genome to describe their characteristics. Here we investigated how the choice of negative training set influences the performance of

a prediction model. We generated gene sets of different sizes randomly sampled from non-HI genes with complete predictor variable information and compared the cross-validation performance (AUC) resulting from the use of these gene sets as the negative training set to the use of the HS gene set as the negative training set (Figure S5). The use of a judiciously selected HS gene set is clearly advantageous.

3.3 Influence of CNV discovery parameters on prediction performance

We further investigated if our model performance is sensitive to CNV discovery and filtering parameters. We examined the influence on cross-validation performance of using different confidence thresholds (Birdseye LOD score) in CNV discovery and population frequency when generating HS gene set. A greater LOD score indicates higher confidence and thus a more stringent CNV set. Similarly, the more frequently a gene is found LOF in apparently healthy individuals, the more likely it is haplosufficient, and thus the negative training set is more stringent. We found that LOD score threshold has little influence on the model performance (Figure S6). The use of recurrent LOF genes exhibits an apparent improvement of performance over the use of all LOF genes under most LOD thresholds. Further increase in stringency by requiring higher frequency results in further reduction of the size of negative training set, but little if any increase in performance of the prediction model. Therefore, we adopted the negative training set generated under LOD > 10 and found in at least two individuals in further analysis.

3.4 Comparing the use of LDA and SVM as classifier

We investigated if the use of support vector machine (SVM), a more sophisticated machine learning method, as classifier would improve prediction performance. An SVM model was trained on the same training set as LDA with optimized parameters ($gamma = 0.1$, $cost = 1$) and class weights. The performance was examined by self-validation, leave-one-out cross-validation and 10-fold cross-validation (Figure S7). Despite being more sophisticated and computational expensive, SVM exhibits no appreciable improvement over LDA (Figure S7).

4 Validation using external data

4.1 Validation using genes implicated in genetic diseases with known mode of inheritance

We tested if genes with higher predicted probability of being haploinsufficient, p(HI), are enriched in genes implicated in dominant disease relative to genes implicated in recessive diseases using Fisher's exact test. To do so, genes were labeled HI or HS by a threshold placed on p(HI). We further investigated how the extent of enrichment varies with changing thresholds. The most significant enrichment was found when genes with the highest 20% of p(HI) were treated as HI genes.

4.2 Validation using orthologs of mouse HI genes

We tested if genes with higher p(HI) are enriched in human orthologs of mouse HI genes and mouse haplolethal genes (HL). Again, we investigate how the extent of enrichment changes over shifting threshold on p(HI). As expected, the enrichment in HL genes is constantly higher than in HI genes, since HL is a more specific and severe phenotype and is strongly opposed to phenotype ('apparently healthy') of the negative training set.

4.3 Validation using putative pathogenic deletions

We tested if our prediction is able to pick out genomic intervals, when deleted, known to cause genomic disorders from intervals that tolerate decreased copy number. The model we adopted considers the phenotypic consequence as the result of the cumulative yet independent effect of all affected genes and the probability of an interval being HI is defined as: $p_{interval}\,(\mathrm{HI}) = 1 - \prod \left(1 - p_{gene}\,(\mathrm{HI})\right)$.[1] We then used the derived LOD score: $LOD = ln\left(\frac{p_{interval}\,(\mathrm{HI})}{1 - p_{interval}\,(\mathrm{HI})}\right)$, as the metric of haploinsufficiency of the genomic interval being assessed. A 'null' distribution of LOD score could be obtained by collecting the maximum LOD score (LOD_{max}) found in each individual in a large apparently healthy cohort, against which the likelihood of a LOF variant being pathogenic can be assessed.

To prevent circularity, we retained a subset (2,322 GWAS controls used in studies of Schizophrenia and Bipolar disease) of the 8,458 apparently healthy individuals from which the HS genes in the original training data were derived and generated a new set of $p_{gene}\,(\mathrm{HI})$ by training on the reduced HS gene set identified from the rest of apparently healthy individuals using the same method as described in section 1. After imputation of predictor variables (see section 5), this new training set contains 287 HI genes and 594 HS genes (234 HI genes and 270 HS genes before imputation). The model trained from this reduced training set achieved a similar AUC and MCC in 10-fold cross-validation as the model trained from the original training set (after imputation: AUC = 0.84, MCC = 0.55; before imputation: AUC = 0.81, MCC = 0.50). The resulting predictions are also highly consistent with the original predictions (correlation between p(HI) is 0.99 both before and after imputation). We used the predictions based on the dataset that includes imputed predictor variables to allow the more reliable assertion of haploinsufficiency of an genomic interval from the vast majority of the genes affected by its deletion. The retained subset of apparently healthy individuals contains 1433 European Americans and 889 African Americans. They together yielded 1607 LOF genes, of which 1031 for which p(HI) can be generated. Population specific summary is listed in Table S4 and distribution of LOD_{max} per individual is shown in Figure 7 of the main text. In general, LOD_{max} per individual and the proportion of individuals with a LOD_{max} in the top 1% of the pooled distribution is higher in European Americans than African Americans, which is consistent to the speculation that purifying selection is more efficient in African populations due to larger effective

[1] $p_{gene}\,(\mathrm{HI})$ is simply referred to as p(HI) in the rest of the document.

population size. However both differences are not statistically significant (p = 0.35, Mann-Whitney U test for max LOD; p = 0.24, Fisher's exact test for proportion in top 1%).

We collected 487 *de novo* deletions identified from array-based CNV detection and classified as being putatively pathogenic in the DECIPHER database [8]. The distribution of LOD score in putatively pathogenic loci compared to LOD_{max} of apparently healthy individuals is shown in Figure 7 of the main text. An example of using LOD score to prioritize candidate-gene-discovery in putatively pathogenic deletions is given in Figure S8.

4.4 Allele frequency spectrum of nonsynonymous variants in genes with different p(HI)

We used the resequencing dataset described in Boyko *et al* [9], which contains 47,576 SNPs found by direct resequencing of 11,404 protein coding genes in 35 individuals (20 European Americans and 15 African Americans), to test whether p(HI) is related to the allele frequency spectrum. We expect that genes under stronger negative selection should exhibit an enrichment of rare alleles in their allele frequency spectrum relative to genes under less selective constraint. There are 14,420 nonsynonymous SNPs and 16,213 synonymous SNPs in the dataset found within genes with predicted p(HI). We examined their derived allele frequency (DAF) spectrum as a function of p(HI) of the genes in which they are located (Figure S9). Regardless of population composition, the DAF spectrum of nonsynonymous SNPs are more skewed towards rare variants in gene sets with higher p(HI) than in those with lower p(HI), indicating greater selective constraint on the genes with higher p(HI). We assessed the significance of this observation using a one-sided Mann-Whitney U test to compare the median of the allele frequency spectrum of nonsynonymous variants in genes with p(HI) in the top 20% with that of nonsynonymous variants in genes with p(HI) in the bottom 80%. The p value for this test in European Americans was 3.95e-3, and in African Americans was 2.85e-7. The p values for the analogous test for synonymous sites in the same populations were 0.127 and 0.0566 respectively.

5 Expanding genomic coverage through imputation

5.1 Pattern of missing data

Before embarking on imputation-based approaches to increase the number of genes for which we can predict their probability of exhibiting haploinsufficiency we examined the distribution of missing data among genes (Table S4). Most genes that do not have complete data for all predictor variables are missing only 1 or 2 variables.

5.2 Multiple imputation

Multiple imputation is an effective method to overcome missing data in multivariable data analysis [10, 11]. The method was used here to fill in ('impute') the missing values for three of the four predictor variables incorporated in the model, namely 'dN/dS ratio between human and macaque', 'promoter conservation (GERP)', and 'gene network proximity to HI genes', except for 'embryonic expression' of which the genomic coverage is 100%. Since 'gene network proximity to HI genes' and 'promoter conservation (GERP)' are the top two predictive variables, genes missing both values were removed. To achieve better imputation, we included three additional gene properties, namely 'CDS conservation (GERP)', 'spliced transcript length' and 'gene network betweenness centrality' in the imputation process. Twenty independent imputations of 20 iterations were undertaken. In each iteration, imputation for each predictor variable was in the order of increasing number of missing values using the predictive mean matching method. The computation was done using the R package MICE [12].

The number of genes without missing values among the six predictor variables increased from 12,443 to 17,456, before and after imputation. This allowed both a larger training set and a higher coverage of the genome from which the likelihood of being haploinsufficient can be predicted.

5.3 Model training and performance

After imputation, the size of training set was enlarged from 234 HI and 326 HS genes to 287 HI and 679 HS genes. An LDA model was trained by this dataset and the model performance assessed by 10-fold cross-validation improved from 0.807 to 0.833. Network proximity to known HI genes was still the most predictive variable (Figure S10).

5.4 Validation using external data

After imputation, we were able to predict the probability of being haploinsufficient for roughly 85% of the genome. We investigated if enrichment of gene sets related to haploinsufficiency could still be observed in this set of predictions of increased genomic coverage.

First, we calculated the fold of enrichment of genes implicated (by OMIM) in human dominant diseases relative to recessive diseases as a function of shifting p(HI) threshold. Enrichment of dominant genes relative to recessive genes was still observed, although the overall extent of enrichment is decreased (Figure S11). Under the threshold where top 10% predictions are labeled as prediction HI genes, there is a 3.19-fold of enrichment (p=1.71e-11, Fisher's exact test). Next, we calculated the fold of enrichment of human orthologs of mouse haploinsufficient genes and haplolethal genes relative to the genome average as a function of shifting p(HI) threshold. Comparable or slightly increased fold of enrichment is observed after imputation when compared to that before imputation (Figure S12). Under the threshold where

top 10% predictions are labeled as predicted HI genes, the enrichment of mouse haploinsufficient genes is 2.57 fold and the enrichment of mouse haplolethal genes is 4.51 fold. Overall, we concluded the quality of prediction on the basis of imputed data was at comparable level with that on the basis of un-imputed data.

References

1. McCarroll SA, Kuruvilla FG, Korn JM, Cawley S, Nemesh J, et al. (2008) Integrated detection and population-genetic analysis of SNPs and copy number variation. Nat Genet 40: 1166-74.

2. International Schizophrenia Consortium (2008) Rare chromosomal deletions and duplications increase risk of schizophrenia. Nature 455: 237-41.

3. Korn J, Kuruvilla F, McCarroll S, Wysoker A, Nemesh J, et al. (2008 Sep 7) Integrated genotype calling and association analysis of SNPs, common copy number polymorphisms and rare CNVs. Nat Genet .

4. Hubbard TJP, Aken BL, Ayling S, Ballester B, Beal K, et al. (2009 Jan) Ensembl 2009. Nucleic Acids Res 37: D690-7.

5. Kondrashov FA, Koonin EV (2004) A common framework for understanding the origin of genetic dominance and evolutionary fates of gene duplications. Trends Genet 20: 287-90.

6. Nguyen DQ, Webber C, Ponting CP (2006) Bias of selection on human copy-number variants. PLoS Genet 2: e20.

7. Dang VT, Kassahn KS, Marcos AE, Ragan MA (2008) Identification of human haploinsufficient genes and their genomic proximity to segmental duplications. Eur J Hum Genet 16: 1350-7.

8. Firth HV, Richards SM, Bevan AP, Clayton S, Corpas M, et al. (2009) DECIPHER: DatabasE of Chromosomal Imbalance and Phenotype in Humans using Ensembl Resources. Am J Hum Genet 84: 524-33.

9. Boyko AR, Williamson SH, Indap AR, Degenhardt JD, Hernandez RD, et al. (2008) Assessing the evolutionary impact of amino acid mutations in the human genome. PLoS Genet 4: e1000083.

10. Donders ART, van der Heijden GJMG, Stijnen T, Moons KGM (2006) Review: a gentle introduction to imputation of missing values. J Clin Epidemiol 59: 1087-91.

11. van der Heijden GJMG, Donders ART, Stijnen T, Moons KGM (2006) Imputation of missing values is superior to complete case analysis and the missing-indicator method in multivariable diagnostic research: a clinical example. J Clin Epidemiol 59: 1102-9.

12. Van Buuren S, Groothuis-Oudshoorn C (2000) Multivariate Imputation by Chained Equations: MICE V1.0 User's manual. Leiden: TNO Preventie en Gezondheid.

Figure legends

Figure S1. Procedure for LOF calling

The flow chart shows the pipeline used to identify LOF genes. A gene with all its transcripts disrupted under any of the four considered LOF scenarios is regarded as LOF. On the right, the numbers under each scenario denotes the number of detected LOF events meeting that criteria. A LOF event is defined as loss of function of one transcript in one individual.

Figure S2. Number of human haplosufficient genes discovered from Affymetrix 6.0 array data

The plot shows the number of LOF genes discovered as a function of the number of apparently healthy individuals being assayed. The red line represents all LOF genes whereas the gene line represents recurrent LOF genes, *i.e.* HS genes.

Figure S3. Comparison of model performance

The AUCs of each combination of predictor variables in 10-fold cross validation repeated 30 times are shown as vertical bars with error bars represent 2 times standard deviation. The mean AUC (red), mean MCC (green) and the overall gene coverage (blue) are labeled on top of each bar. The bar pointed by the black arrow head is the chosen combination of predictor variables.

Figure S4. Prediction performance of single predictor variable and integrated model

Mean AUC of each model in 10-fold cross-validation repeated 30 times are shown as vertical bars with the actual values label at the top.

Figure S5. Prediction performance of using HS and genome background as negative training set

The plot compares the cross-validation performances resulted from using different gene sets as negative training set. The triangle represents HS gene set generated from CNV data. The squares represent different sizes of random gene sets sampled from the genome after excluding known HI genes. For each size,

the gene set was sampled 20 times and the standard deviation of the resulting performances is shown as error bar.

Figure S6. Prediction performance under different parameters used in generation of negative training set

The cross-validation performance (AUC) resulted from using negative training sets generated with different parameters are represented by blue vertical bars with axis on the left. The sizes of these negative training sets are represented by red vertical bars with axis on the right. Bars are grouped by the CNV calling parameters, LOD score, and within each group the darkness of coloring represent different frequency threshold used to define HS as shown in the legend. The bar pointed by the black arrow head represent parameters and corresponding negative training set adopted in further analysis.

Figure S7. Comparing the prediction performance of LDA and SVM

The plot shows the comparison of prediction performance between LDA (dark bar) and SVM (light bar) using three approaches (from left to right): self-validation, leave-one-out cross-validation and 10-fold cross-validation. In the first two comparisons, SVM exhibits only very marginal improvement over LDA, whereas in the third LDA is marginally better.

Figure S8. Examples of highlighting candidate genes, the 8p23.1 deletion

GATA4, the gene whose haploinsufficiency is attributed to the congenital heart malformation phenotype of the 8p23.1 deletion syndrome, is shown in this screenshot of the DECIPHER web browser to have the highest predicted haploinsufficiency of all 24 genes in this 3.4 Mb deletion interval.

Figure S9. Derived allele frequency spectrum of variants in different gene sets

This figure shows the spectrum of derived allele frequency (DAF, represented here as counts of derived allele in the population) of nonsynonymous SNPs and synonymous SNPs discovered by resequencing of human genes in **a)** 15 African Americans and **b)** 20 European Americans. In each plot, DAF of variants located in genes of different p(HI) are compared side by side, where bars of decreasing darkness represent quantiles of decreasing p(HI), such that the 0∼25% quartile is that with the highest probability of being

haploinsufficient.

Figure S10. Assessment of model performance after imputation

The ROC curve demonstrates the performance of the model trained on the enlarged training set using 10-fold cross-validation. The error bars represent standard errors of the mean. The lower right inset shows the relative contribution of each predictor variable to the prediction model measured by the absolute value of the scaling factor of each predictor variable constituting the linear discriminant.

Figure S11. Enrichment of predicted HI genes in dominant genes relative to recessive genes

The plot compares the fold of enrichment of predicted HI genes in dominant genes relative to recessive genes before (red line and circle) and after (blue line and triangle) imputation. under a shifting threshold of p(HI) above which genes are regarded as HI.

Figure S12. Enrichment of predicted HI genes in orthologs of mouse haploinsufficient genes and mouse haplolethal genes

The plot compares the fold of enrichment of predicted HI genes in human orthologs of mouse haploinsufficient genes (red lines) and mouse haplolethal genes (blue lines) relative to the genome average before (darker lines with squares) and after (lighter lines with triangles) imputation under a shifting threshold of p(HI) above which genes are regarded as HI.
